# Comprehensive assessment of the expression of the SWI/SNF complex defines two distinct prognostic subtypes of ovarian clear cell carcinoma

**DOI:** 10.18632/oncotarget.10181

**Published:** 2016-06-20

**Authors:** Hisham Abou-Taleb, Ken Yamaguchi, Noriomi Matsumura, Ryusuke Murakami, Hidekatsu Nakai, Koichiro Higasa, Yasuaki Amano, Kaoru Abiko, Yumiko Yoshioka, Junzo Hamanishi, Masafumi Koshiyama, Tsukasa Baba, Ryo Yamada, Fumihiko Matsuda, Ikuo Konishi, Masaki Mandai

**Affiliations:** ^1^ Department of Gynecology and Obstetrics, Graduate School of Medicine, Kyoto University, Kyoto, Japan; ^2^ Department of Obstetrics and Gynecology, Faculty of Medicine, Assiut University, Assiut, Egypt; ^3^ Department of Obstetrics and Gynecology, Kinki University Faculty of Medicine, Osaka-Sayama, Japan; ^4^ Center for Genomic Medicine, Graduate School of Medicine, Kyoto University, Kyoto, Japan

**Keywords:** clear cell carcinoma, ovarian cancer, SWI/SNF complex, copy number variation

## Abstract

Somatic mutations in the *ARID1A* tumor-suppressor gene have been frequently identified in ovarian clear cell carcinoma (CCC) cases. BAF250a encoded by *ARID1A* is a member of the SWI/SNF complex, but the expression and mutation status of other SWI/SNF subunits have not been explored. The current study aimed to elucidate the biological and clinical significance of the SWI/SNF complex subunits, by assessing the expression and mutation status of SWI/SNF subunits, and distinct genomic aberrations associated with their expression. Of 82 CCC specimens, 38 samples presented no BAF250a expression, and 50 samples exhibited the loss of at least one subunit of the SWI/SNF complex. Cases which lack at least one SWI/SNF complex component exhibited significantly more advanced stages, faster growth and stronger nuclear atypia compared with SWI/SNF-positive samples (p<0.05). Although BAF250a expression is not related to poor prognosis, the group presenting the loss of at least one SWI/SNF complex subunit exhibited significantly shorter overall and progression-free survivals (p<0.05). A multivariate analysis suggested that the expression status of the SWI/SNF complex serves as an independent prognostic factor (p<0.005). The cases positive for all SWI/SNF subunits demonstrated significantly greater DNA copy number alterations, such as amplification at chromosomes 8q.24.3 and 20q.13.2-20q.13.33 (including *ZNF217*) and deletion at chromosomes 13q12.11-13q14.3 (including *RB1*), 17p13.2-17p13.1 (including *TP53*) and 19p13.2-19p13.12. In conclusion, the CCCs exhibiting the loss of one or multiple SWI/SNF complex subunits demonstrated aggressive behaviors and poor prognosis, whereas the CCCs with positive expression for all SWI/SNF components presented more copy number alterations and a favorable prognosis.

## INTRODUCTION

Ovarian cancer is the most devastating gynecological malignancy in the world and is a heterogeneous disease with distinct clinicopathological and molecular features [1, 2]. The pathological classification of epithelial ovarian cancers includes four major histological subtypes based entirely on tumor cell morphological criteria: serous carcinoma (SC), mucinous carcinoma (MC), endometrioid carcinoma (EC) and clear cell carcinoma (CCC). Of these, CCC is one of the most aggressive types because unlike high-grade SC, it is refractory to conventional cytotoxic chemotherapeutic agents [3]. Recent genome-wide studies in ovarian CCC have identified somatic mutations in AT-rich interactive domain 1A (*ARID1A*) in 46-57% of ovarian CCC cases [4, 5] and also identified unique DNA methylation profiles, characterized by hypomethylation of the hepatocyte nuclear factor-1-beta (*HNF1B*) pathway and hypermethylation of the estrogen receptor alpha (*ERα*) pathway [6]. BAF250a encoded by *ARID1A* is a member of the SWItch/Sucrose Non-Fermentable (SWI/SNF) chromatin remodeling complex, which comprises polymorphic assemblies of at least 14 subunits encoded by 28 genes, generating an extensive diversity of complexes with specialized functions in specific tissues [7–10]. Although several subunits of the SWI/SNF complex have been reported to possess tumor-suppressive functions in the malignancies of several organs [7–9], the expression and mutation status of SWI/SNF complex subunits, with the exception of BAF250a, have yet to be explored in ovarian CCC. The impact of BAF250a on clinical prognosis is controversial [11, 12]. Furthermore, the biological and functional roles of the SWI/SNF complex in ovarian CCC have yet to be elucidated. In this study, the expression of SWI/SNF complex subunits and BAF250a was assessed to explore the biological and clinical significance of these proteins in ovarian CCC.

## RESULTS

### Expression status of the SWI/SNF complex in different histological subtypes of ovarian cancer

**Figure 1:**
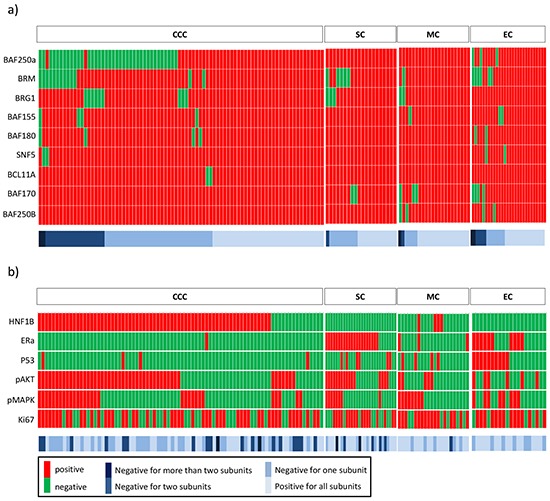
Expression heatmaps **a.** Expression of nine subunits belonging to the SWI/SNF complex. The color bar represents the number of subunits that demonstrate a loss of expression. Dark blue indicates that the sample lacks more than two subunits. Blue indicates that the sample is negative for two subunits. Light blue indicates that the sample is negative for one subunit. The lightest blue indicates that the sample expresses all subunits. **b.** Expression of six molecules that represent biological features. The red columns represent positive expression, whereas the green columns represent negative expression.

Representative immunohistochemistry results for nine SWI/SNF complex subunits (BAF250a, BAF250B encoded by *ARID1B*, BRM encoded by *SMARCA2*, BRG1 encoded by *SMARCA4*, BAF155 encoded by *SMARCC1*, BAF170 encoded by *SMARCC2*, SNF5 encoded by *SMARCB1*, BCL11A and BAF180 encoded by *PBRM1*) are shown in Supplementary Figure S1. The expression status of each SWI/SNF complex subunit is shown in Figure 1-a, and the frequency of loss of each subunit is summarized in Supplementary Figure S2 and Supplementary Table S1. The most frequently lost subunit of the SWI/SNF complex was BAF250a (41/152, 27.0%). Interestingly, BAF250a was lost in CCC (38/82, 46.3%) and, to a lesser extent, in EC (3/28, 10.7%), whereas none of the SC or MC cases showed loss of BAF250a expression (p<0.0001, Supplementary Figure S2-a and Supplementary Table S1).

Conversely, the BAF250a complementary subunit, BAF250B, continued to be expressed in all ovarian cancer cases with the exception of two EC and two MC cases, indicating that this subunit is one of the less frequently lost subunits. The other frequently lost subunits were SNF5 and BCL11A, which were lost in four and two cases, respectively (Figure 1, Supplementary Figures S2-g, S2-h and S2-i, and Supplementary Table S1). BAF170 exhibited positive expression in all CCC cases, whereas 11.5% of the non-CCC samples exhibited no BAF170 expression, indicating a statistically significant difference (p<0.0088, Supplementary Figure S2-d and Supplementary Table S1).

### Distinct molecular characteristics of different histological subtypes of ovarian cancer

The expression of six molecules related to tumor development (HNF1B, ERα, P53, pAKT, pMAPK and Ki-67) was analyzed through immunohistochemistry, as shown in Supplementary Figure S3, and the expression status of each is summarized in Figure 1-b and Supplementary Figure S4.

A significantly larger proportion of the 67 CCC cases (82%) was positive for HNF1B immunostaining, whereas the other cancer types with the exception of four MC cases were all negative for HNF1B (p<0.0001, Figure 1-b and Supplementary Figure S4-a). ERα expression was lost in all CCC cases with the exception of one, whereas ERα continued to be expressed in 75% of EC (21/28), 50% of SC and 17% of MC cases (p<0.0001, Figure 1-b and Supplementary Figure S4-b). P53 accumulated (Immuno-Reactive Score, IRS≥150) in 50% of SC, 22% of EC and 18% of MC cases, whereas out of the 82 tested CCC cases, only four cases had an elevated IRS for P53 (p<0.0001, Figure 1-b and Supplementary Figure S4-c). The highest percentage of AKT phosphorylation, denoting PIK3CA/AKT/mTOR pathway activation, was observed in CCC (61%). pAKT expression in EC reached 57% positivity, and pAKT expression in both CCC and EC was significantly higher than that in SC (40%) and MC (22%) (p<0.0001, Figure 1-b and Supplementary Figure S4-d). pMAPK expression in the four subtypes was almost within the same 40% range (p=0.9035, Figure 1-b and Supplementary Figure S4-e).

High proliferation indices (≥25%) were detected in 62.2%, 60.7%, 70% and 72.7% of CCC, EC, SC and MC cases, respectively. The comparison of the proliferation index values among the different types revealed that CCC presents significantly slower proliferation than the other three subtypes (p=0.0116, Supplementary Figure S4-f).

### Expression of SWI/SNF complex subunits in CCC

The most frequently lost subunit in CCC was BAF250a (38/82, 46.3%), followed by BRM (13/82, 15.9%) and BRG1 (9/82, 11%). Few cases showed negative reactions with BAF155, BAF180, SNF5 and BCL11A, whereas BAF250Band BAF170 appeared to be positively stained in all CCC cases (Figure 1-a). Of the 82 CCC cases, 50 samples (61.0%) exhibited the loss of at least one SWI/SNF complex subunit. Thirty-one cases (37.8%) lost immunoreactivity for one subunit only, whereas 19 cases (23.2%) lacked at least two subunits. Of the 19 cases that showed a loss of expression of multiple subunits, 15 cases demonstrated the loss of BAF250a together with either BRM or BRG1 (Figure 1-a). The two cases that lacked SNF5 also presented a loss of expression of BRM, whereas the two cases in which BCL11A was lost did not lack any other SWI/SNF subunits.

### Effect of the loss of SWI/SNF complex subunits on CCC

Whether CCC cases in which SWI/SNF complex expression was lost presented certain clinical, biological, pathological or molecular features that may distinguish these cases from CCC cases in which SWI/SNF expression is preserved was then investigated.

**Figure 2:**
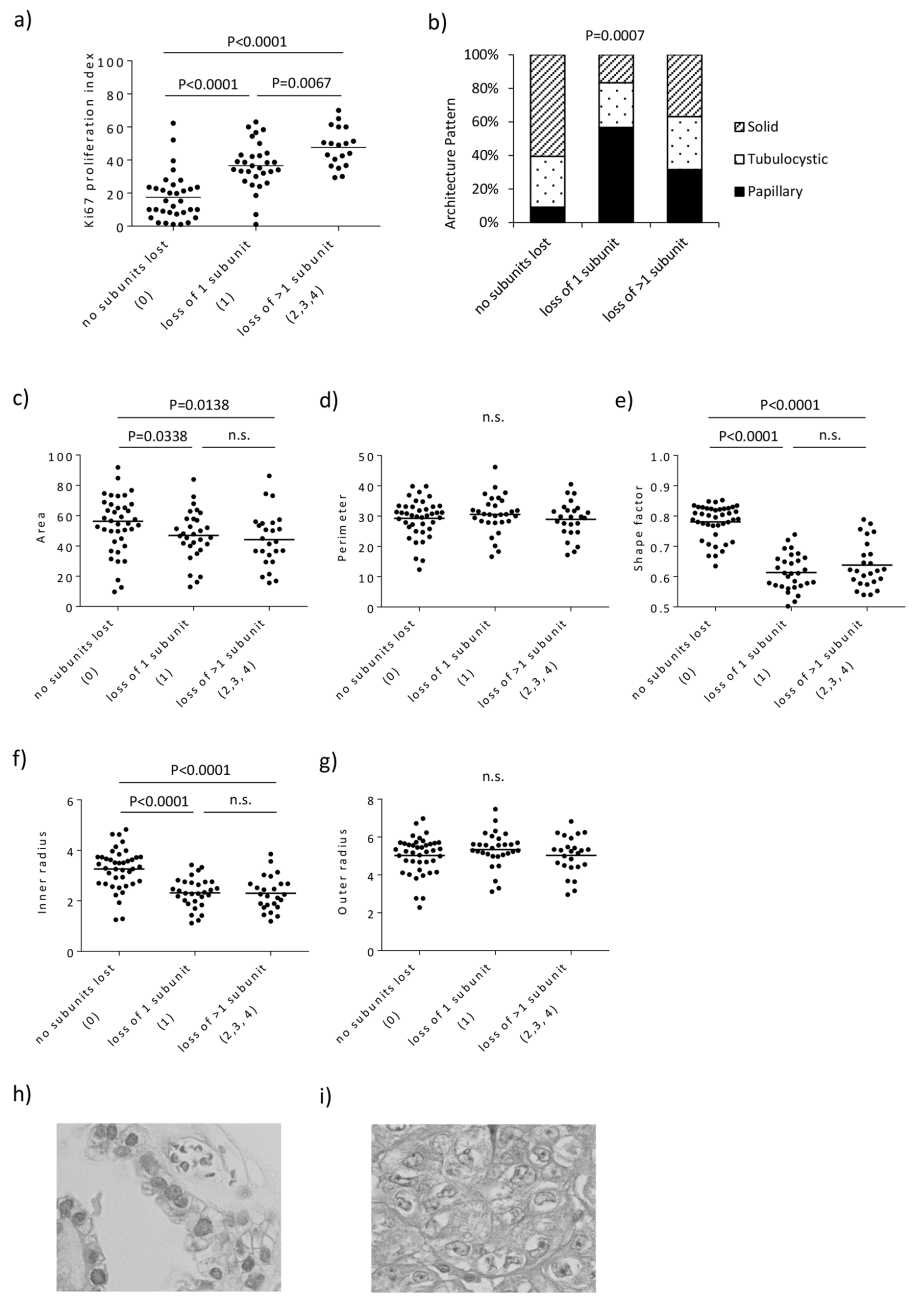
Distinctive proliferation and morphological features based on SWI/SNF complex expression status **a.** Cases lacking multiple subunits had the highest Ki-67 proliferation indices. **b.** Loss of SWI/SNF subunit expression in ovarian clear cell carcinoma tended to result in papillary architectural patterns. **c-g.** The cases lacking at least one SWI/SNF component had smaller and flatter nuclei with irregular outlines. The nuclei in the SWI/SNF-positive group are more rounded and symmetrical and showed no beading in the nuclear membrane **h.** whereas more elongation, beading and knobbing of the nuclear outline, as well as breaks in the nuclear membrane, were observed in cases lacking at least one subunit **i**.

First, the clinical backgrounds of the cases were assessed. Although there is no association between SWI/SNF complex status and age, tumor size, the presence of endometriosis or body mass index, cases in which SWI/SNF complex expression was lost were at a significantly more advanced stage compared with cases demonstrating positive expression of all SWI/SNF complex subunits (p=0.0094, Table 1). Second, the comparison of the expression status of HNF1B, ERα, P53, pAKT and pMAPK with SWI/SNF subunit staining did not reveal any significant variations between the cases with positive SWI/SNF expression and cases that lacked at least one SWI/SNF component (Supplementary Figures S5-a to S5-e). Third, the influence of the expression of this complex on proliferation was assessed. Cases lacking at least one SWI/SNF component, as well as BAF250a, proliferated more rapidly than positive cases, as determined by comparing their Ki-67 indices (p<0.0001, Figure 2-a and Supplementary Figure S5-f). In addition, cases lacking multiple subunits presented a significantly higher proliferation index than the positive cases (p<0.0001) and the cases lacking a single subunit (p=0.0067), suggesting that the loss of multiple subunits exerted a cumulative effect. Cases presenting the loss of core subunits (BAF250a, BAF250B, BRG1 and BRM) showed significantly higher Ki-67 indices compared with the cases with all core subunits (p<0.0001, Supplementary Figure S5-g). Cases lacking non-core subunits (BAF155, BAF170, BAF180, SNF5 and BCL11A) exhibited higher Ki-67 indices than those with all subunits, but this difference was not significant (p=0.0700).

**Table 1:** The clinical backgrounds among SWI/SNF complex status in CCC

		SWI/SNF positive expression	Loss of expression of one subunit	Loss of expression of multiple subunits	P value
**Age (median, in years)**		51.10	51.75	54.10	0.4854^1^
**FIGO Stage**	Stage I	28	19	6	**0.0094^2^
	Stage II	2	2	2	
	Stage III	1	6	8	
	Stage IV	2	3	3	
**Maximum Tumor diameter (median, in cm)**		11	12	12	0.4158^1^
**Endometriosis**		30/33 (90.91%)	27/30 (90%)	17/19 (89.47%)	0.9844^1^
**BMI (median)**		19.58	21.14	21.88	0.2246^2^

1 P values were calculated using Kruskal Wallis test

2 P values were calculated using Chi-square test

** P value <0.005

The morphology was then evaluated. Because CCC presents three main architectural patterns, specifically papillary, tubulocystic and solid (Supplementary Figure S6), the architectural patterns of the cases were assessed according to the SWI/SNF complex status. The results showed that the architectural patterns varied significantly with respect to changes in SWI/SNF immunoreactivity (p=0.0007, Figure 2-b). The papillary pattern was more prevalent in cases lacking a single subunit, whereas the solid pattern was more common in SWI/SNF-positive cases. Cases lacking multiple subunits could show any of the different patterns, although slightly more cases exhibited the papillary pattern. Because CCC cells frequently exhibit nuclear atypia, the cancer cell nuclei were assessed based on the SWI/SNF status (Supplementary Results). The nuclei of the cells in the SWI/SNF-positive group were found to be more rounded and symmetrical, with no beading and no breaks in the nuclear membrane (Supplementary Figure S7-l), whereas elongation, beading and knobbing of the nuclear surface, along with breaks in the nuclear membrane, were observed in cases lacking at least one SWI/SNF component (Supplementary Figure S7-m).

A microarray analysis identified 58 probes that were overexpressed in cases lacking at least one SWI/SNF component relative to the positive group (Supplementary Table S2). A categorical analysis using DAVID produced 37 terms that were significantly enriched in the cases lacking at least one SWI/SNF complex subunit (Supplementary Table S3). Fourteen of the 37 terms were associated with the cell cycle and proliferation.

These results suggest that the loss of SWI/SNF complex expression contributes to proliferation and nuclear atypia.

### The SWI/SNF complex status in ovarian CCC is an independent prognostic factor

**Figure 3:**
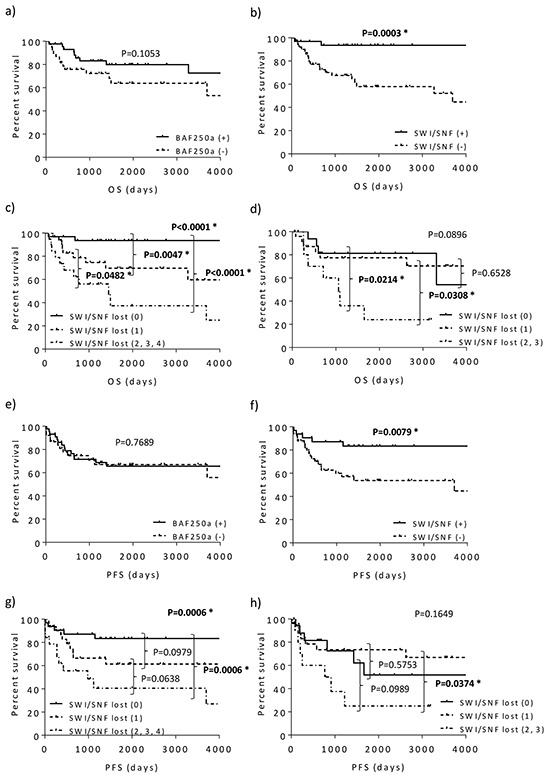
Kaplan-Meier curves for overall (a-d) and progression-free survival (e-h) based on BAF250a status and SWI/SNF complex expression The analysis of the samples from Kyoto University revealed that BAF250a expression is not related to prognosis in terms of overall (a) and progression-free (e) survival, whereas the loss of SWI/SNF subunit expression is associated with poorer prognosis in terms of overall (b) and progression-free (f) survival. The poor prognosis was more evident with the loss of multiple SWI/SNF subunits (overall and progression-free survival, (c) and (g), respectively). The analysis of the validation datasets from Kinki University also indicated that increases in the number of SWI/SNF subunits that presented a loss in expression resulted in poorer prognosis (overall and progression-free survival, (d) and (h), respectively).

First, 82 CCC samples at Kyoto University were used to produce Kaplan-Meier curves. The prognoses of the BAF250a-negative CCC cases in terms of overall survival were not different compared with that of the BAF250a-positive cases (p=0.1053, Figure 3-a). However, cases lacking at least one SWI/SNF complex subunit presented significantly poorer prognosis compared with cases that were positive for all subunits (p=0.0003, Figure 3-b). Furthermore, when divided into three groups, denoted “specifically positive for all SWI/SNF complex subunits”, “negative for one subunit” and “negative for multiple subunits”, the former group presented the most favorable prognosis, whereas the group lacking multiple subunits had the worst prognosis (p<0.0001 for the three groups, Figure 3-c). In terms of progression-free survival, although the BAF250a-negative cases did not demonstrate a significant reduction in survival compared with the BAF250a-positive cases (p=0.7689, Figure 3-e), cases lacking at least one SWI/SNF complex subunit exhibited a significantly poorer prognosis compared with cases in which all SWI/SNF complex subunits were present (p=0.0079, Figure 3-f). Poor prognosis was more evident in cases lacking multiple subunits (p=0.0006 for the three groups, p=0.0006 between the cases positive for all subunits and the cases lacking multiple subunits, p=0.0638 between the cases lacking one subunit and the cases lacking multiple subunits; Figure 3-g). The division of the group of cases lacking at least one subunit into cases lacking core subunits and cases lacking non-core subunits revealed that both groups showed significantly poorer prognosis in terms of overall and progression-free survival compared with the group of cases positive for all subunits (Supplementary Figure S8).

Second, to validate these results at Kyoto University, another set of 54 CCC samples from Kinki University was stained to assess their SWI/SNF complex status. Interestingly, similar results were obtained: a loss of expression resulted in a worse prognosis in terms of both overall and progression-free survival, and this result was more marked with the loss of multiple subunits compared with the loss of one subunit (p<0.05 for both, Figures 3-d and 3-h).

To identify prognostic factors for ovarian CCC, univariate and multivariate analyses of the overall and progression-free survival rates were performed using the combined datasets from Kyoto and Kinki Universities. The univariate analysis of overall survival showed that the loss of expression of at least one SWI/SNF subunit, FIGO staging, residual tumor size and complication with thromboembolism were significant prognostic factors for CCC survival (p=0.0005, p<0.0001, p<0.0001 and p=0.0022, respectively; Table 2). The multivariate analyses indicated that the loss of expression of at least one SWI/SNF subunit in addition to the FIGO stage was an independent prognostic factor associated with poor overall survival (p=0.0132 and p=0.0030, respectively; Table 2). For progression-free survival, the loss of expression of multiple SWI/SNF subunits, FIGO staging, residual tumor size and complication with thromboembolism were significant prognostic factors (p=0.0038, p<0.0001, p<0.0001 and p=0.0008, respectively; Table 3). The FIGO stage was identified as an independent prognostic factor for progression-free survival (p=0.0001, Table 3). To evaluate the impact of the loss of multiple subunits, a Cox proportional hazards regression model was implemented using the following two groups: the cases lacking no or a single SWI/SNF component and the group presenting the loss of multiple SWI/SNF complex subunits. The loss of multiple SWI/SNF subunits, as well as the FIGO stage, were found to be independent prognostic factors associated with poor overall survival (p=0.0005 and p=0.0042, respectively; Supplementary Table S4) and progression-free survival (p=0.0030 and p<0.0001, respectively; Supplementary Table S5). These findings suggest that the loss of SWI/SNF complex subunits is a prognostic factor, and the strength of this factor is more evident with the loss of multiple subunits.

**Table 2:** Univariate and multivariate analysis of overall survival rate

OS			Univariate Analysis				Multivariate Analysis			
Variable	subsets	N	HR	95% CI		P value	HR	95% CI		P value
				Lower	Upper			Lower	Upper	
**Age**	<55y	75	1.3233	0.7066	2.4785	0.3815				
	≥55y	61								
**FIGO stage**	I & II	88	6.0998	3.0999	12.0026	^****^<0.0001	1.9191	1.2483	2.9502	**0.0030
	III & IV	48								
**Residual tumor**	Complete excision	109	4.4910	2.4173	8.3438	^****^<0.0001	1.1818	0.8027	1.7400	0.3974
	Residual tumor >1cm	27								
**Tumor Size**	<10cm	42	1.7761	0.8166	3.8632	0.1474				
	≥10cm	94								
**Thromboembolism**	Absence	121	3.2576	1.5294	6.9389	**0.0022	1.2887	0.8648	1.9204	0.2128
	Presence	15								
**SWI/SNF complex**	Number of negative subunit (0)	51	5.3290	2.0865	13.6107	***0.0005	3.3790	1.2903	8.8490	**0.0132
	Number of negative subunit (1, 2, 3)	85								
**BAF250a**	Positive expression	75	1.3472	0.7233	2.5092	0.3477				
	Negative expression	61								

Univariate and Multivariate analysis were done using Cox Regression Model

* p<0.05

** p<0.005

*** p <0.0005

**** p<0.0001

**Table 3:** Univariate and multivariate analysis of progression-free survival rate

PFS			Univariate Analysis				Multivariate Analysis			
Variable	subsets	N	HR	95% CI		P value	HR	95% CI		P value
				Lower	Upper			Lower	Upper	
**Age**	<55y	75	1.1550	0.6399	2.0850	0.6325				
	≥55y	61								
**FIGO stage**	I & II	88	7.4504	3.8828	14.2962	^****^<0.0001	2.2256	1.4802	3.3464	***0.0001
	III & IV	48								
**Residual tumor**	Complete excision	109	4.9298	2.7104	8.9666	^****^<0.0001	1.1832	0.8284	1.6898	0.3550
	Residual tumor >1cm	27								
**Tumor Size**	<10cm	42	1.3966	0.6913	2.8214	0.3519				
	≥10cm	94								
**Thromboembolism**	Absence	121	3.3814	1.6579	6.8963	**0.0008	1.2549	0.8662	1.8180	0.2300
	Presence	15								
**SWI/SNF complex**	Number of negative subunit (0)	51	2.9455	1.4175	6.1203	**0.0038	1.6640	0.7762	3.5672	0.1906
	Number of negative subunit (1, 2, 3)	85								
**BAF250a**	Positive expression	75	0.9241	0.5113	1.6701	0.7938				
	Negative expression	61								

Univariate and Multivariate analysis were done using Cox Regression Model

* p<0.05

** p<0.005

*** p <0.0005

**** p<0.0001

### Mutational status of the SWI/SNF complex subunits and copy number variations in CCC

Exome sequencing was performed to identify single nucleotide variants (SNVs) of genes belonging to the SWI/SNF complex and to evaluate genome-wide copy number variations (CNVs) in the expression of SWI/SNF complex subunits. SNVs were identified in four genes (*ARID1A*, *SMARCD3*, *ARID1B* and *BCL11B*; Supplementary Figure S9). Somatic mutations occurred most frequently in *ARID1A*, with a frequency of 75.0% (12 out of 16). The second most frequent SNV was in *ARID1B* (12.5%, two out of 12), followed by *BCL11B* and *SMARCD3* (6.3%, one out of 12 for both).

The SNV status was compared with protein immunoreactivity in 16 samples that included both tumor specimens and normal counterparts (Supplementary Figure S10). Nine out of 12 samples that had mutations in *ARID1A* exhibited no BAF250a expression, whereas two out of four cases that did not have mutations in *ARID1A* exhibited positive expression for BAF250a (sensitivity = 75.0% and specificity = 50.0%, Supplementary Table S6). Immunohistochemistry analysis showed that two cases that possessed mutations in the *ARID1B* gene expressed the BAF250B protein. No SNVs were identified in the other seven genes.

**Figure 4:**
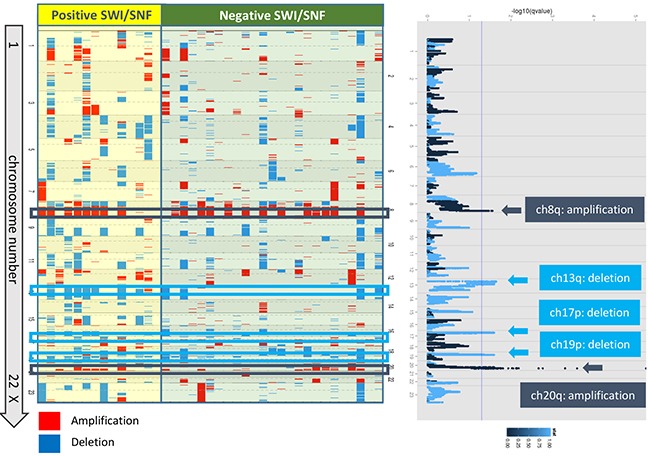
Copy number alterations according to chromosomal loci Left) DNA copy number changes are represented as pseudo-color gradients corresponding to the copy number increase (red boxes) and decrease (blue boxes). Each column represents an individual tumor sample. Right) Copy number aberrations for the SWI/SNF-positive cases compared with the negative cases are depicted on Manhattan plots. The dark and light blue colors indicate more amplification and deletion in the positive cases compared with the negative cases.

To elucidate genomic differences defined by CNVs, 14 cases that showed positive expression for all SWI/SNF complex subunits were compared with 25 samples that demonstrated the loss of expression of at least one subunit. The positive cases presented increased amplification of chromosomes 8q.24.3 and 20q.13.2-20q.13.33 (including *ZNF217*) and increased deletion of chromosomes 13q12.11-13q14.3 (including *RB1*), 17p13.2-17p13.1 (including *TP53*) and 19p13.2-19p13.12 (Figure 4). Therefore, the SWI/SNF-positive group presented more frequent chromosomal alterations compared with the cases lacking at least one SWI/SNF subunit.

## DISCUSSION

The impact on clinical behaviors and the expression and mutation status of SWI/SNF complex subunits have yet to be explored in ovarian CCC. Loss of BAF250a expression was found to be specific to CCC (46.3% of CCC specimens) in this study. This result is compatible with those obtained in previous studies, which showed that BAF250a expression is lost in 42% of ovarian CCC cases [4]. The analysis of the nine SWI/SNF subunits revealed that 61% of CCC samples lacked the expression of at least one subunit and that 23.2% of CCC cases exhibited the loss of multiple SWI/SNF subunits, suggesting a cumulative loss of SWI/SNF complex expression. However, this study has some limitations. One limitation is that other SWI/SNF components, such as BAF200 encoded by *ARID2*, BCL11B, and BRD9, were not evaluated. Another is that some variants cannot be detected by immunohistochemistry. A comprehensive assessment and a knowledge-based analysis are necessary to elucidate the expression of the entire SWI/SNF complex in ovarian CCC.

Mutations in the *ARID1A* and *TP53* genes are mutually exclusive, and BAF250a requires the formation of a complex with P53 to inhibit P53-related genes, including tumor suppressors, in gynecologic malignancies [13]. Our findings showed no relationship between SWI/SNF complex expression and P53 expression in CCC samples. One possible reason is the distinct evaluation methods used in our investigation compared with those used in a previous study. Specifically, immunoreactivity was assessed in this study, whereas Guan et al. evaluated the mutation status of *ARID1A* and *TP53* [13]. Another reason is the discrepancy between P53 protein expression and *TP53* gene mutations [14]. Although the Ki-67 index indicated a slow growth of ovarian CCC compared with the other histological subtypes, the absence of SWI/SNF subunits, as well as BAF250a, was significantly correlated with advanced stages and proliferation of ovarian CCC, supporting the role of the SWI/SNF complex as a tumor-suppressive complex. The present study also indicated that the absence of SWI/SNF complex expression is correlated with the architectural papillary pattern and nuclear irregularity. Bourgo et al. showed that loss of BRG1 leads to nuclear malformations [15]. Imbalzano et al. demonstrated that *BRG1* knockdown induces grooves in the nuclear periphery through internal nuclear mechanisms [16]. Nuclear atypia, which is often observed in ovarian CCC cells, likely results from loss of SWI/SNF complex function. However, Bennett et al. reported that grading ovarian clear cell carcinomas based on architectural patterns and nuclear shape does not provide survival differences, indicating that the clinical significance of these factors is controversial [17, 18]. In fact, our samples did not show that the architectural pattern serves as a prognostic factor (data not shown). The impact of the SWI/SNF status on nuclear atypia requires further investigation because the evaluation of nuclear atypia is not definitive.

Katagiri et al. reported that loss of BAF250a expression is correlated to shorter progression-free survival, whereas Maeda et al. insisted that there are no significant differences between BAF250a-positive and BAF250a-negative cases [11, 12]. In our study, BAF250a expression, as a single factor, did not contribute to overall and progression-free survival; however, the expression of SWI/SNF complex subunits was an independent prognostic factor of ovarian CCC. This phenomenon became more evident with the loss of multiple subunits, suggesting that the function of the SWI/SNF complex, as well as that of BAF250a, is cumulative and essential for tumor progression in CCC cases. Because the results obtained using the CCC samples from Kyoto University were validated using ovarian CCC samples from Kinki University, these reproducible findings using independent datasets indicate that the expression of the entire SWI/SNF complex is a useful biomarker for predicting the prognosis of ovarian CCC.

Although genome-wide analyses conducted by Kadoch et al. identified several mutations in SWI/SNF complex subunits. [7], these genes have been identified in only a limited number of samples. In the current study, 16 CCC samples, which is a limited number of samples, were used to identify mutations in SWI/SNF complex subunits. Four genes, including two novel genes, associated with the SWI/SNF complex were found to be mutated in ovarian CCC. *ARID1A* was the most frequently mutated gene in the SWI/SNF complex, and its expression was found to be negatively correlated with its mutational status. However, the analysis of the correlation of *ARID1A* expression with its mutational status in five of the samples introduced discrepancies. For these heterozygous cases, Weigand et al. reasoned a detectable protein that is produced by some mutations and post-transcriptional or post-translational regulation or dominant-negative effects of the mutations [4]. Three cases investigated in this study that showed positive immunoreactivity for BAF250a instead of harboring mutations showed unknown amino acid alterations even if the sequence exhibited frameshift insertion or deletion mutations. For the other subunits, with the exception of BAF250a, our findings suggest that the expression is not suppressed by mutations, which may be due to the limited number of samples used for the comparison of the mutational status with protein expression. Thus, additional cases should be studied to resolve the discrepancy between expression and mutational status. Another reason for this discrepancy could be that the expression of SWI/SNF subunits is suppressed by microRNA and methylation in malignancies of several organs [19–24]. In this study, we detected amplification at chromosomes 8q and 20q in many CCC samples. Kuo et al. found that the level of chromosomal instability in CCC, as defined by the extent of DNA copy number alterations, is similar to that observed in low-grade ovarian SC but markedly less than that found in high-grade SC, and the most remarkable aberration is a gain at chromosome 20q [25]. Rahman et al. also reported chromosome 20q locus amplification in ovarian CCC [26]. Two previous studies demonstrated the gain of chromosomes 1q, 8q, 17q and 20q and the loss of chromosomes 8p, 9q and 19p in ovarian CCC [27, 28]. Our data are compatible with these previous reports. The comparison of CNV with the SWI/SNF complex status revealed amplifications at chromosomes 8q.24.3 and 20q.13.2-20q.13.33 and deletions at chromosomes 13q12.11-13q14.3, 17p13.2-17p13.1 and 19p13.2-19p13.12 in the cases that retained the expression of all subunits compared with the cases that lacked SWI/SNF subunits. However, no specific copy number change was observed in the group that presented a loss of SWI/SNF expression. The chromosome 20q13.2 includes *ZNF217*, which was reported to be a frequently amplified locus and related to cell growth and anti-apoptosis in CCC [25, 26]. Chromosome 13q14.2 comprises *RB1*, and chromosome 17p13.1 is composed of *TP53*. The deletion of *RB1* and *TP53* is commonly observed in somatic copy number analyses across different types of cancer [29]. Positive protein expression of SWI/SNF and copy number alterations appear to be inversely correlated. Although the mechanism through which an intact SWI/SNF complex is associated with copy number alterations has not yet been elucidated, a similar phenomenon was observed in a different malignancy. *ARID5B* classified in the SWI/SNF complex family is less mutated in the high-copy-number subtype of endometrial carcinoma [30]. These findings suggest that SWI/SNF-positive cases are characterized by genomic instability with frequent copy number aberrations [2, 31, 32].

In summary, the absence of at least SWI/SNF complex subunit as well as BAF250a activates proliferation and irregularities in the nuclear shape. The loss of SWI/SNF complex subunits is a prognostic factor, and this finding is more evident with the loss of multiple subunits. BAF250a expression is suppressed by mutations, which are most frequently found in this subunit among the SWI/SNF subunits. Two subtypes of ovarian CCC can be classified based on SWI/SNF complex expression. The first subtype includes cases in which the expression of SWI/SNF complex subunits is lost and is associated with poor prognosis due to aggressive growth. The second subtype includes cases that fully express the SWI/SNF complex. The genetic background of this subtype involves more frequent copy number alterations relative to cases lacking at least one subunit. These findings may lead to the development of novel diagnostic tools and therapeutic strategies based on precision medicine.

## MATERIALS AND METHODS

Refer to Supplementary Materials and Methods.

### Tissue materials

This study was approved by the Institutional Review Board of Kyoto University and Kinki University. One hundred and fifty-two epithelial ovarian cancer cases comprising 82 CCC, 28 EC, 20 SC and 22 MC cases were investigated to determine the expression of nine main SWI/SNF complex subunits (BAF250a, BAF250B, BRM, BRG1, BAF155, BAF170, SNF5, BCL11A and BAF180). Another 54 CCC samples from Kinki University were used to validate the clinical significance of the expression of these nine SWI/SNF complex subunits. Staining for HNF1B, ERα, P53, pAKT, pMAPK and Ki-67 was also performed. All of the antibodies are listed in Supplementary Table S7.

### Pathological review

Clear cell carcinomas exhibit three main architectural patterns, namely papillary, tubulocystic and solid patterns [33, 34]. The tumor pattern is assigned based on the predominance of a certain architecture in more than 50% of all examined sections of the tumor. The nuclear shape was analyzed using the integrated morphometry package of MetaMorph^®^ Image analyzer (version 6.1, Universal Imaging Corp., West Chester, PA, USA).

### Evaluation of staining

Protein expression was evaluated by determining the Immuno-Reactive Score (IRS) [35], which is obtained by multiplying the percentage of positively stained cells by the intensity of the reaction. The percentage of positive cells ranged from 0% to 100%, and the intensity of the reaction was scored a value from 0 to 3 as follows: no reaction, 0; weak, 1; moderate, 2; or strong, 3. Therefore, the scores ranged from 0 to 300. Scores between 0 and 49 were classified as negative, whereas scores ≥50 were considered to indicate positive expression.

In the analysis of P53 staining, cases with high immunoreactivity scores (≥150) were considered to suggest a potential gain-of-function mutation of *TP53*, whereas cases with lower scores were considered to express wild-type P53 or null mutations based on a previously published report [14]. The IRSs were reviewed by a gynecologic pathologist who was blinded to the patient data.

The Ki-67 labeling index was analyzed using image analysis software (ImageJ) [36]. For each case, 10 high-power field areas (×400) of maximal tumor positivity were selected, and each field contained 50-250 nuclei. The percentages of positive nuclei were determined [37].

### Microarray analysis

A total of 17 ovarian CCC samples, which were frozen immediately after surgery and stored at −80°C, were used for mRNA expression microarray analysis. Microarray analysis was performed using Affymetrix Human Genome U133A 2.0 Arrays following standard Affymetrix protocols (Affymetrix, Santa Clara, CA, USA). Fifty-eight genes were identified as differentially expressed between cases lacking at least one SWI/SNF component and positive cases if the expression change was greater than twofold based on a t-test with a p-value less than 0.05. The biological roles of the 58 genes were analyzed by categorical analysis (gene ontology terms and TRANSFAC, which represents activation of transcription) using the web-based Database for Annotation, Visualization and Integrated Discovery (DAVID) v6.7 (http://david.abcc.ncifcrf.gov/).

### Whole-exome sequencing

The frozen tissue samples of 39 CCC cases and 16 lymphocyte samples isolated from whole blood of the corresponding CCC patients were used for whole-exome sequencing analyses. To identify somatic mutations (single nucleotide variants), 16 samples were compared with their normal counterparts. Thirty-nine CCC cases were assessed for copy number variations between SWI/SNF-positive cases and cases lacking one or more SWI/SNF subunits. GISTIC2.0 [38] and the Bioconductor package “copynumber” in R [39] were used for the analysis and visualization of the copy number variations (CNVs). A samroc analysis [40] was performed to evaluate the copy numbers between SWI/SNF-positive CCC cases and CCC cases lacking one or more subunits.

## SUPPLEMENTARY RESULTS



## References

[1] Cho KR, Shih I-M (2009). Ovarian Cancer. Annual Review of Pathology-Mechanisms of Disease.

[2] Yamaguchi K, Matsumura N, Mandai M, Baba T, Konishi I, Murphy SK (2014). Epigenetic and genetic dispositions of ovarian carcinomas. Oncoscience.

[3] Sugiyama T, Kamura T, Kigawa J, Terakawa N, Kikuchi Y, Kita T, Suzuki M, Sato I, Taguchi K (2000). Clinical characteristics of clear cell carcinoma of the ovary: a distinct histologic type with poor prognosis and resistance to platinum-based chemotherapy. Cancer.

[4] Wiegand KC, Shah SP, Al-Agha OM, Zhao Y, Tse K, Zeng T, Senz J, McConechy MK, Anglesio MS, Kalloger SE, Yang W, Heravi-Moussavi A, Giuliany R, Chow C, Fee J, Zayed A (2010). ARID1A mutations in endometriosis-associated ovarian carcinomas. N Engl J Med.

[5] Jones S, Wang TL, Shih Ie M, Mao TL, Nakayama K, Roden R, Glas R, Slamon D, Diaz LA, Vogelstein B, Kinzler KW, Velculescu VE, Papadopoulos N (2010). Frequent mutations of chromatin remodeling gene ARID1A in ovarian clear cell carcinoma. Science.

[6] Yamaguchi K, Huang Z, Matsumura N, Mandai M, Okamoto T, Baba T, Konishi I, Berchuck A, Murphy SK (2014). Epigenetic determinants of ovarian clear cell carcinoma biology. Int J Cancer.

[7] Kadoch C, Hargreaves DC, Hodges C, Elias L, Ho L, Ranish J, Crabtree GR (2013). Proteomic and bioinformatic analysis of mammalian SWI/SNF complexes identifies extensive roles in human malignancy. Nat Genet.

[8] Wilson BG, Roberts CW (2011). SWI/SNF nucleosome remodellers and cancer. Nat Rev Cancer.

[9] Hohmann AF, Vakoc CR (2014). A rationale to target the SWI/SNF complex for cancer therapy. Trends Genet.

[10] Ho L, Crabtree GR (2010). Chromatin remodelling during development. Nature.

[11] Katagiri A, Nakayama K, Rahman MT, Rahman M, Katagiri H, Nakayama N, Ishikawa M, Ishibashi T, Iida K, Kobayashi H, Otsuki Y, Nakayama S, Miyazaki K (2012). Loss of ARID1A expression is related to shorter progression-free survival and chemoresistance in ovarian clear cell carcinoma. Mod Pathol.

[12] Maeda D, Mao TL, Fukayama M, Nakagawa S, Yano T, Taketani Y, Shih Ie M (2010). Clinicopathological Significance of Loss of ARID1A Immunoreactivity in Ovarian Clear Cell Carcinoma. Int J Mol Sci.

[13] Guan B, Wang TL, Shih IM (2011). ARID1A, a factor that promotes formation of SWI/SNF-mediated chromatin remodeling, is a tumor suppressor in gynecologic cancers. Cancer Res.

[14] Ho ES, Lai CR, Hsieh YT, Chen JT, Lin AJ, Hung MH, Liu FS (2001). p53 mutation is infrequent in clear cell carcinoma of the ovary. Gynecol Oncol.

[15] Bourgo RJ, Siddiqui H, Fox S, Solomon D, Sansam CG, Yaniv M, Muchardt C, Metzger D, Chambon P, Roberts CW, Knudsen ES (2009). SWI/SNF deficiency results in aberrant chromatin organization, mitotic failure, and diminished proliferative capacity. Mol Biol Cell.

[16] Imbalzano KM, Cohet N, Wu Q, Underwood JM, Imbalzano AN, Nickerson JA (2013). Nuclear shape changes are induced by knockdown of the SWI/SNF ATPase BRG1 and are independent of cytoskeletal connections. PLoS One.

[17] Bennett JA, Dong F, Young RH, Oliva E (2015). Clear cell carcinoma of the ovary: evaluation of prognostic parameters based on a clinicopathological analysis of 100 cases. Histopathology.

[18] Yamamoto S, Tsuda H, Shimazaki H, Takano M, Yoshikawa T, Kuzuya K, Kurachi H, Kigawa J, Kikuchi Y, Sugiyama T, Matsubara O (2012). Histological grading of ovarian clear cell adenocarcinoma: proposal for a simple and reproducible grouping system based on tumor growth architecture. Int J Gynecol Pathol.

[19] Wang N, Zhou Y, Zheng L, Li H (2014). MiR-31 is an independent prognostic factor and functions as an oncomir in cervical cancer via targeting ARID1A. Gynecol Oncol.

[20] Khursheed M, Kolla JN, Kotapalli V, Gupta N, Gowrishankar S, Uppin SG, Sastry RA, Koganti S, Sundaram C, Pollack JR, Bashyam MD (2013). ARID1B, a member of the human SWI/SNF chromatin remodeling complex, exhibits tumour-suppressor activities in pancreatic cancer cell lines. Br J Cancer.

[21] Skulte KA, Phan L, Clark SJ, Taberlay PC (2014). Chromatin remodeler mutations in human cancers: epigenetic implications. Epigenomics.

[22] Song S, Walter V, Karaca M, Li Y, Bartlett CS, Smiraglia DJ, Serber D, Sproul CD, Plass C, Zhang J, Hayes DN, Zheng Y, Weissman BE (2014). Gene silencing associated with SWI/SNF complex loss during NSCLC development. Mol Cancer Res.

[23] Wang L, Zhao Z, Meyer MB, Saha S, Yu M, Guo A, Wisinski KB, Huang W, Cai W, Pike JW, Yuan M, Ahlquist P, Xu W (2014). CARM1 methylates chromatin remodeling factor BAF155 to enhance tumor progression and metastasis. Cancer Cell.

[24] Sakurai K, Furukawa C, Haraguchi T, Inada K, Shiogama K, Tagawa T, Fujita S, Ueno Y, Ogata A, Ito M, Tsutsumi Y, Iba H (2011). MicroRNAs miR-199a-5p and -3p target the Brm subunit of SWI/SNF to generate a double-negative feedback loop in a variety of human cancers. Cancer Res.

[25] Kuo KT, Mao TL, Chen X, Feng Y, Nakayama K, Wang Y, Glas R, Ma MJ, Kurman RJ, Shih IM, Wang TL (2010). DNA copy numbers profiles in affinity-purified ovarian clear cell carcinoma. Clin Cancer Res.

[26] Rahman MT, Nakayama K, Rahman M, Nakayama N, Ishikawa M, Katagiri A, Iida K, Nakayama S, Otsuki Y, Shih IM, Miyazaki K (2012). Prognostic and therapeutic impact of the chromosome 20q13. 2 ZNF217 locus amplification in ovarian clear cell carcinoma. Cancer.

[27] Hirasawa A, Saito-Ohara F, Inoue J, Aoki D, Susumu N, Yokoyama T, Nozawa S, Inazawa J, Imoto I (2003). Association of 17q21-q24 gain in ovarian clear cell adenocarcinomas with poor prognosis and identification of PPM1D and APPBP2 as likely amplification targets. Clin Cancer Res.

[28] Sung CO, Choi CH, Ko YH, Ju H, Choi YL, Kim N, Kang SY, Ha SY, Choi K, Bae DS, Lee JW, Kim TJ, Song SY, Kim BG (2013). Integrative analysis of copy number alteration and gene expression profiling in ovarian clear cell adenocarcinoma. Cancer Genet.

[29] Zack TI, Schumacher SE, Carter SL, Cherniack AD, Saksena G, Tabak B, Lawrence MS, Zhsng CZ, Wala J, Mermel CH, Sougnez C, Gabriel SB, Hernandez B, Shen H, Laird PW, Getz G (2013). Pan-cancer patterns of somatic copy number alteration. Nat Genet.

[30] Kandoth C, Schultz N, Cherniack AD, Akbani R, Liu Y, Shen H, Robertson AG, Pashtan I, Shen R, Benz CC, Yau C, Laird PW, Ding L, Zhang W, Mills GB, Kucherlapati R (2013). Integrated genomic characterization of endometrial carcinoma. Nature.

[31] Ciriello G, Miller ML, Aksoy BA, Senbabaoglu Y, Schultz N, Sander C (2013). Emerging landscape of oncogenic signatures across human cancers. Nat Genet.

[32] Kuo KT, Guan B, Feng Y, Mao TL, Chen X, Jinawath N, Wang Y, Kurman RJ, Shih IM, Wang TL (2009). Analysis of DNA copy number alterations in ovarian serous tumors identifies new molecular genetic changes in low-grade and high-grade carcinomas. Cancer Res.

[33] DeLair D, Oliva E, Kobel M, Macias A, Gilks CB, Soslow RA (2011). Morphologic spectrum of immunohistochemically characterized clear cell carcinoma of the ovary: a study of 155 cases. The American journal of surgical pathology.

[34] Okamoto A, Glasspool RM, Mabuchi S, Matsumura N, Nomura H, Itamochi H, Takano M, Takano T, Susumu N, Aoki D, Konishi I, Covens A, Ledermann J, Mezzanzanica D, Mezzazanica D, Steer C (2014). Gynecologic Cancer InterGroup (GCIG) consensus review for clear cell carcinoma of the ovary. Int J Gynecol Cancer.

[35] Halon A, Materna V, Drag-Zalesinska M, Nowak-Markwitz E, Gansukh T, Donizy P, Spaczynski M, Zabel M, Dietel M, Lage H, Surowiak P (2011). Estrogen receptor alpha expression in ovarian cancer predicts longer overall survival. Pathology oncology research : POR.

[36] Schneider CA, Rasband WS, Eliceiri KW (2012). NIH Image to ImageJ: 25 years of image analysis. Nature methods.

[37] Wilson GD, Saunders MI, Dische S, Daley FM, Robinson BM, Martindale CA, Joiner B, Richman PI (1996). Direct comparison of bromodeoxyuridine and Ki-67 labelling indices in human tumours. Cell proliferation.

[38] Mermel CH, Schumacher SE, Hill B, Meyerson ML, Beroukhim R, Getz G (2011). GISTIC2. 0 facilitates sensitive and confident localization of the targets of focal somatic copy-number alteration in human cancers. Genome Biol.

[39] Nilsen G, Liestøl K, Van Loo P, Moen Vollan HK, Eide MB, Rueda OM, Chin SF, Russell R, Baumbusch LO, Caldas C, Børresen-Dale AL, Lingjaerde OC (2012). Copynumber: Efficient algorithms for single- and multi-track copy number segmentation. BMC Genomics.

[40] Broberg P (2003). Statistical methods for ranking differentially expressed genes. Genome Biol.

