# Supplementary material for: Comprehensive assessment of the expression of the SWI/SNF complex defines two distinct prognostic subtypes of ovarian clear cell carcinoma
Source: Oncotarget. 2016 Jun 20;7(34):54758–70. doi: 10.18632/oncotarget.10181 (PMC5342379; doi:10.18632/oncotarget.10181)
Supplement: Supplementary file 1 [file oncotarget-07-54758-s001.pdf]

Comprehensive assessment of the expression of the SWI/SNF complex defines two distinct prognostic subtypes of ovarian clear cell carcinoma

SUPPLEMENTARY RESULTS

Effect of the loss of SWI/SNF complex subunits on nuclear shape in CCC

Because CCC cells frequently exhibit nuclear atypia, the cancer cell nuclei were assessed based on their SWI/SNF status. The total area of the nucleus was smaller in cases lacking at least one SWI/SNF component compared with the nuclear area in the positive cases ($p<0.05$, Supplementary Figure S7-a); however, the perimeter of the nucleus was not significantly different ($p=0.5712$, Supplementary Figure S7-b). This difference may be due to the marked irregularity in the nuclear shape of the cells in the cases lacking at least one SWI/SNF subunit. The roundness, denoted by the shape factor, is markedly reduced in negative cells compared with immunoreactive cells ($p<0.0001$, Supplementary Figure S7-c). Direct observations (Supplementary Figures S7-l and S7-m) revealed many nuclei with grooves and invaginations of the nuclear membrane, resulting in significant reductions in the inner radii ($p<0.0001$, Supplementary Figure S7-d) without significant changes to the outer radii of the nuclei ($p=0.4529$, Supplementary Figure S7-e). Other parameters associated with nuclear shape did not vary among the CCC groups (Supplementary Figures S7-f, S7-g, S7-h, S7-i, S7-j and S7-k). These results suggest that the nuclei of the cells in the SWI/SNF-positive group are more rounded and symmetrical, with no beading and no breaks in the nuclear membrane (Supplementary Figure S7-l), whereas elongation, beading and knobbing of the nuclear surface, along with breaks in the nuclear membrane, were observed in cases lacking at least one SWI/SNF component (Supplementary Figure S7-m).

SUPPLEMENTARY MATERIALS AND METHODS

Tissue materials

This study was approved by the Institutional Review Board of Kyoto University and Kinki University. Surgical specimens from 152 cases of epithelial ovarian cancer were selected from cases treated via surgical resection from 1982 to 2014 in the Department of Gynecology and Obstetrics, Kyoto University. All of the patients provided informed consent. The 152 cases, which comprised 82 CCC, 28 EC, 20 SC and 22 MC cases, were investigated to determine the expression of nine main SWI/SNF complex subunits (BAF250a, BAF250B, BRM, BRG1, BAF155, BAF170, SNF5, BCL11A and BAF180). Another 54 CCC samples from Kinki University were used to validate the clinical significance of the expression of these nine SWI/SNF complex subunits. Staining for HNF1B, ERα, P53, pAKT, pMAPK and Ki-67 was also performed. All of the antibodies are listed in Supplementary Table S7.

Pathological review

All tumor samples were independently reviewed by a gynecologic pathologist who was blinded to the patient data. Cases in which the review diagnosis differed from the original diagnosis were further reviewed by a second gynecologic pathologist. The tumors were histologically classified according to World Health Organization (WHO) criteria [1].

Clear cell adenocarcinomas exhibit three main architectural patterns: papillary, tubulocystic and solid patterns [2]. The papillary pattern is characterized by papillae that contain fibrous tissue or hyaline material, whereas the tubulocystic pattern is characterized by multiple tubules and cysts that are variable in size and lined by cuboidal to flattened epithelium. The solid pattern consists of groups of polyhedral cells with abundant clear cytoplasm separated by delicate fibrovascular stroma. The tumor pattern is assigned based on the predominance of a certain architecture in more than 50% of all examined sections of the tumor.

Immunohistochemistry

Nine proteins that compose the SWI/SNF complex and six proteins related to tumor development were stained to allow comparisons of their expression profiles in CCC with their expression profiles in other histological subtypes of ovarian cancer.

Sections from formaldehyde-fixed, paraffin-embedded tissue were deparaffinized and rehydrated. Antigen retrieval was performed by placing sections in citrate buffer (pH 6.0) and then in a decloaking chamber at 120°C for 10 minutes. Heat-induced epitope retrieval (HIER) of BAF180, SNF5, HNF1B and pAKT was performed with EDTA buffer (pH

8.0). After endogenous peroxidase activity was blocked, the sections were sequentially incubated with 5% normal blocking serum for 30 minutes, the appropriate antibodies (Supplementary Table S7), the corresponding biotinylated anti-mouse, anti-goat or anti-rabbit IgG (Nichirei Histofine® SAB-PO staining kit, Nichirei Biosciences, Tokyo, Japan) and an avidin–biotin peroxidase complex from the same kit for 30 minutes. The samples were then exposed to diaminobenzidine tetrahydrochloride solution (DAKO® DAB Chromogen Tablets in D_2W, Dako, Glostrup, Denmark).

Evaluation of staining

Protein expression was evaluated by determining the Immuno-Reactive Score (IRS) [3], which is obtained by multiplying the percentage of positively stained cells by the intensity of the reaction. The percentage of positive cells ranged from 0% to 100%, and the intensity of the reaction was given a score ranging from 0 to 3 as follows: no reaction, 0; weak, 1; moderate, 2; or strong, 3. Therefore, the scores ranged from 0 to 300. Scores between 0 and 49 were classified as negative, whereas scores ≥50 were considered to indicate positive expression.

In the analysis of P53 staining, cases with high immunoreactivity scores (≥150) are considered to exhibit a potential gain-of-function mutation of *TP53*, whereas cases with lower scores are considered to express wild-type P53 or null mutations based on the results from a previous study [4]. The IRSs were reviewed by a gynecologic pathologist who was blinded to the patient data.

The Ki-67 labeling index was analyzed using image analysis software (ImageJ) [5]. For each case, 10 high-power field areas (×400) of maximal tumor positivity were selected, and each field contained 50-250 nuclei. The percentages of positive nuclei were determined [6]. For the clinicopathologic correlations, Ki-67 positivity was classified into two groups: the low-proliferation group contained less than 25% Ki-67-positive tumor cells, and the high-proliferation group contained at least 25% Ki-67-positive tumor cells [7].

Analysis of nuclear shape

Several images were captured for CCC hematoxylin and eosin staining at high magnification (100× oil-immersion lens using a Nikon DS-Fi1 digital camera coupled to a Nikon Eclipse Ti microscope [Nikon, Tokyo, Japan] and a 40× lens using a Bravio 9000 [KEYENCE, Osaka, Japan]). The nuclear shape was analyzed using the integrated morphometry package of the MetaMorph® Image analyzer (version 6.1, Universal Imaging Corp., West Chester, PA, USA). The images were converted to 16-bit monochrome images, and the tumor cell nuclei were selected as regions of interest (ROIs) to measure different parameters of nuclear morphology (Supplementary Figure S11). At least four areas were selected for each case.

The total area, perimeter (P), shape factor, inner radius, outer radius, and other factors were measured using the imaging software for each thresholded nucleus in the image. The measurements were converted to micrometers after image calibration. The perimeter of the nucleus was measured from the mid-point of each pixel composing the thresholded border or margin. The shape factor has a value from 0 to 1 that corresponds to how closely the region resembles a circle. A value near 0 indicates a flattened object, whereas a value of 1.0 indicates a perfectly circular nucleus. The inner radius is the distance from the centroid to the nearest point along the object's edge. The outer radius is the distance from the centroid to the farthest point along the object's edge. The remaining parameters were defined according to the manufacturer's user guide.

Microarray analysis

A total of 17 ovarian CCC samples, which were frozen immediately after surgery and stored at -80 ºC, were used for mRNA expression microarray analysis. Total RNA was extracted using the Qiagen RNeasy Mini Kit (Qiagen K.K.). Microarray analysis was performed using Affymetrix Human Genome U133A 2.0 Arrays following standard Affymetrix protocols (Affymetrix, Santa Clara, CA, USA). The expression intensities across the samples were normalized using the Robust Multichip Average (RMA) algorithm [8]. Fifty-eight genes were identified as differentially expressed between cases lacking at least one SWI/SNF component and positive cases if the expression change was greater than twofold based on a t-test with a p-value less than 0.05. The biological roles of the 58 genes were analyzed by categorical analysis (gene ontology terms and TRANSFAC, which represents activation of transcription) using the web-based Database for Annotation, Visualization and Integrated Discovery (DAVID) v6.7 (http://david.abcc.ncifcrf.gov/).

Whole-exome sequencing

The frozen tissue samples of 39 CCC cases and 16 lymphocyte samples isolated from whole blood of the corresponding CCC patients were used for whole-exome sequencing analyses. To identify somatic mutations (single nucleotide variants), 16 samples were compared with their normal counterparts. Thirty-nine CCC cases were assessed for copy number variations between SWI/SNF-positive cases and cases lacking at least one SWI/SNF subunit. The detailed methodology is described in a paper describing a genome-wide assessment in ovarian CCC (submitted). GISTIC2.0 [9] and the Bioconductor package "copynumber" in R [10] were used for the analysis and visualization of the copy number variations (CNVs). A samroc analysis [11] was performed

to evaluate the copy numbers between SWI/SNF-positive CCC cases and CCC cases lacking one or more subunits. This analysis was performed using transformed log 2 ratios of tumor/normal coverages (the details of which have been described in a secondary supporting manuscript, currently in submission). The samroc q-values, generated by analyzing the distribution of the false-discovery proportion (using the Benjamini-Hochberg procedure with 100 sampling iterations), were less than 0.05 by intent.

REFERENCES

1. Fattaneh A. Tavassoli PD: Pathology and genetics of tumours of the breast and female genital organs. In: *World Health Organization Classification of tumours.* Edited by (IARC) IAfroC: IARC press; 2003.
2. DeLair D, Oliva E, Kobel M, Macias A, Gilks CB, Soslow RA: Morphologic spectrum of immunohistochemically characterized clear cell carcinoma of the ovary: a study of 155 cases. *The American journal of surgical pathology* 2011, 35:36-44.
3. Halon A, Materna V, Drag-Zalesinska M, Nowak-Markwitz E, Gansukh T, Donizy P, Spaczynski M, Zabel M, Dietel M, Lage H *et al*: Estrogen receptor alpha expression in ovarian cancer predicts longer overall survival. *Pathology oncology research: POR* 2011, 17:511-518.
4. Ho ES, Lai CR, Hsieh YT, Chen JT, Lin AJ, Hung MH, Liu FS: p53 mutation is infrequent in clear cell carcinoma of the ovary. *Gynecologic oncology* 2001, 80:189-193.
5. Schneider CA, Rasband WS, Eliceiri KW: NIH Image to ImageJ: 25 years of image analysis. *Nature methods* 2012, 9:671-675.
6. Wilson GD, Saunders MI, Dische S, Daley FM, Robinson BM, Martindale CA, Joiner B, Richman PI: Direct comparison of bromodeoxyuridine and Ki-67 labelling indices in human tumours. *Cell proliferation* 1996, 29:141-152.
7. Anttila M, Kosma VM, Ji H, Wei-Ling X, Puolakka J, Juhola M, Saarikoski S, Syrjanen K: Clinical significance of alpha-catenin, collagen IV, and Ki-67 expression in epithelial ovarian cancer. *Journal of clinical oncology: official journal of the American Society of Clinical Oncology* 1998, 16:2591-2600.
8. Irizarry RA, Hobbs B, Collin F, Beazer-Barclay YD, Antonellis KJ, Scherf U, Speed TP: Exploration, normalization, and summaries of high density oligonucleotide array probe level data. *Biostatistics* 2003, 4:249-264.
9. Mermel CH, Schumacher SE, Hill B, Meyerson ML, Beroukhim R, Getz G: GISTIC2.0 facilitates sensitive and confident localization of the targets of focal somatic copy-number alteration in human cancers. *Genome Biol* 2011, 12:R41.
10. Nilsen G, Liestøl K, Van Loo P, Moen Vollan HK, Eide MB, Rueda OM, Chin SF, Russell R, Baumbusch LO, Caldas C *et al*: Copynumber: Efficient algorithms for single- and multi-track copy number segmentation. *BMC Genomics* 2012, 13:591.
11. Broberg P: Statistical methods for ranking differentially expressed genes. *Genome Biol* 2003, 4:R41.

SUPPLEMENTARY FIGURES AND TABLES

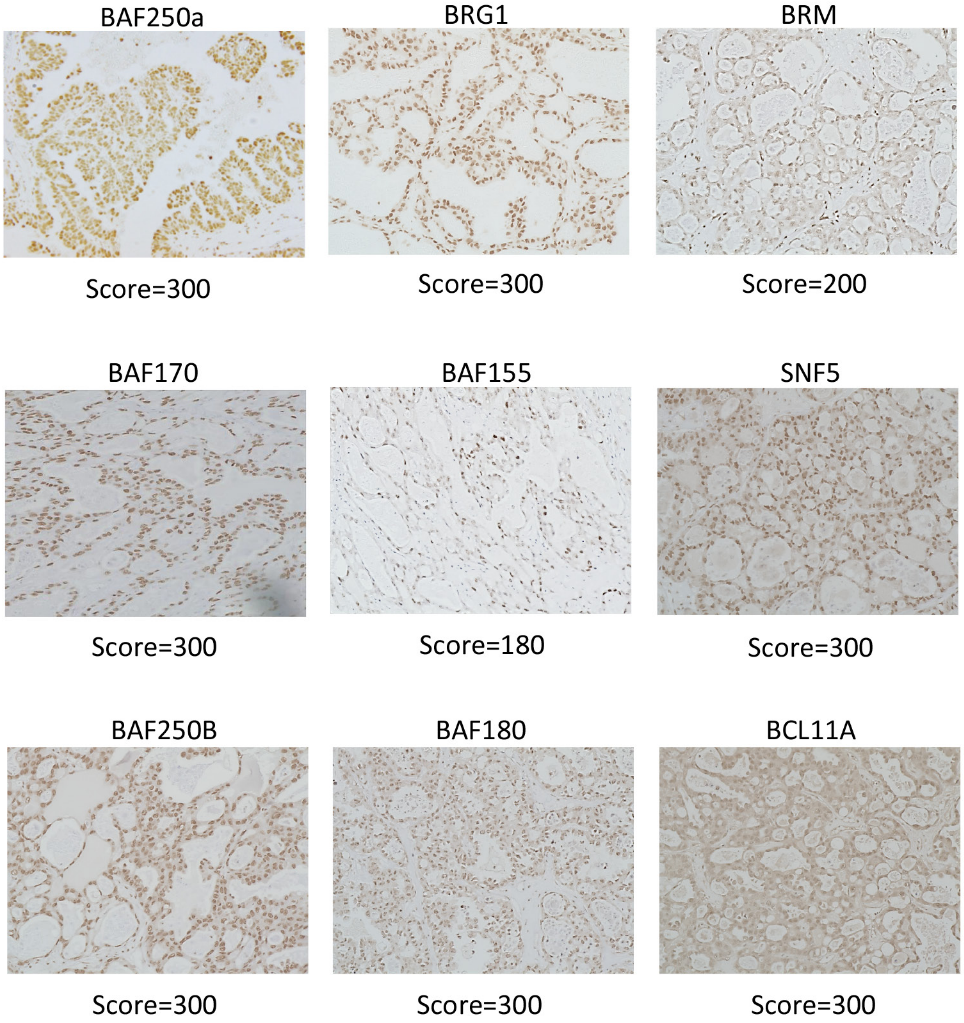

Supplementary Figure S1: Representative immunoreactivity of nine proteins in the SWI/SNF complex.

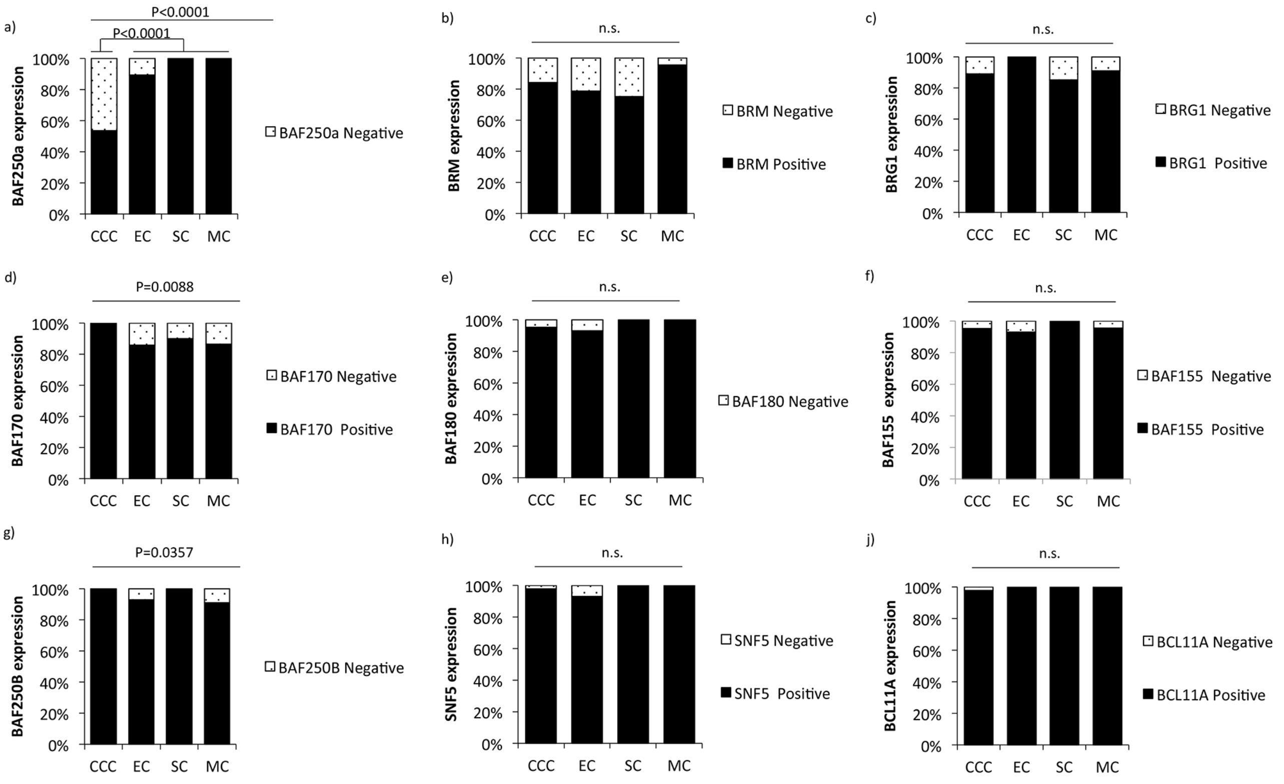

Supplementary Figure S2: Expression status of each protein among the four main histopathological subtypes of epithelial ovarian cancer. a. BAF250a, **b.** BRM, **c.** BRG1, **d.** BAF170, **e.** BAF180, **f.** BAF155, **g.** BAF250B, **h.** SNF5 and **i.** BCL11A. Black bars represent positive expression, whereas dotted bars represent a loss of expression.

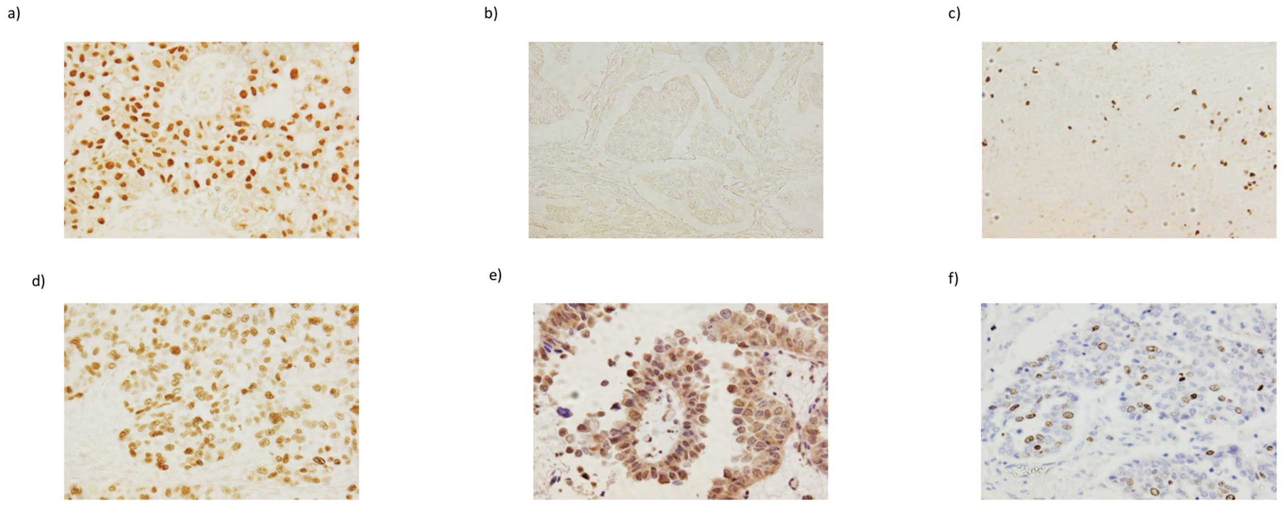

Supplementary Figure S3: Representative figures showing the expression of the following molecules: a. HNF1B, **b.** ERα, **c.** P53, **d.** pMAPK, **e.** pAKT and **f.** Ki-67.

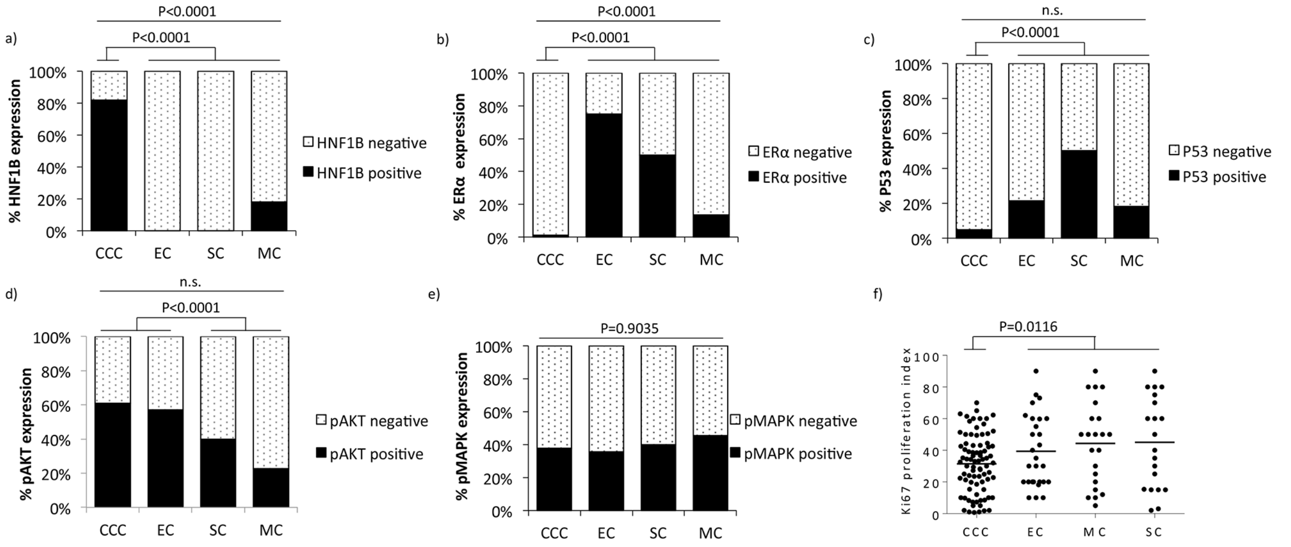

Supplementary Figure S4: Expression status of the tested key molecules among the four main histopathological subtypes of epithelial ovarian cancer. a. HNF1B, **b.** ERα, **c.** P53, **d.** pMAPK, **e.** pAKT and **f.** Ki-67. Solid black bars represent positive expression, and dotted bars represent a loss of expression (a-e). The bars represent the mean Ki-67 index (f).

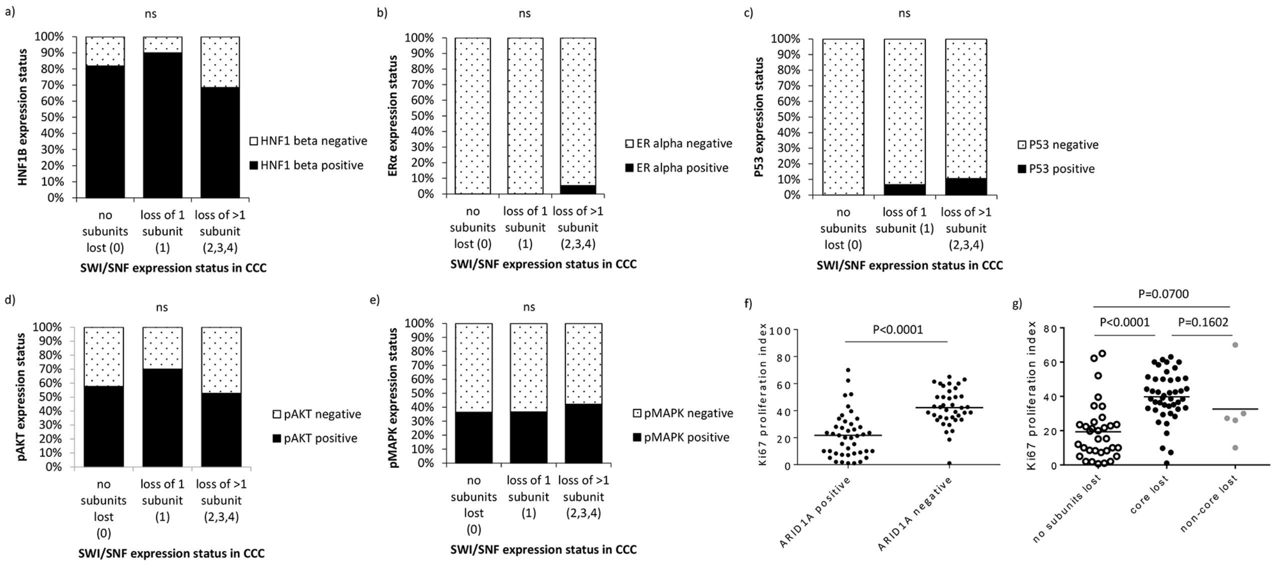

Supplementary Figure S5: Relationship between SWI/SNF complex subunit expression and CCC biomolecular characteristics. a. HNF1B, **b.** ERα, **c.** P53, **d.** pMAPK, **e.** pAKT and **f, g.** Ki-67. Solid black bars represent positive expression, and dotted bars represent a loss of expression (a-e). Bars represent the mean Ki-67 index (f and g).

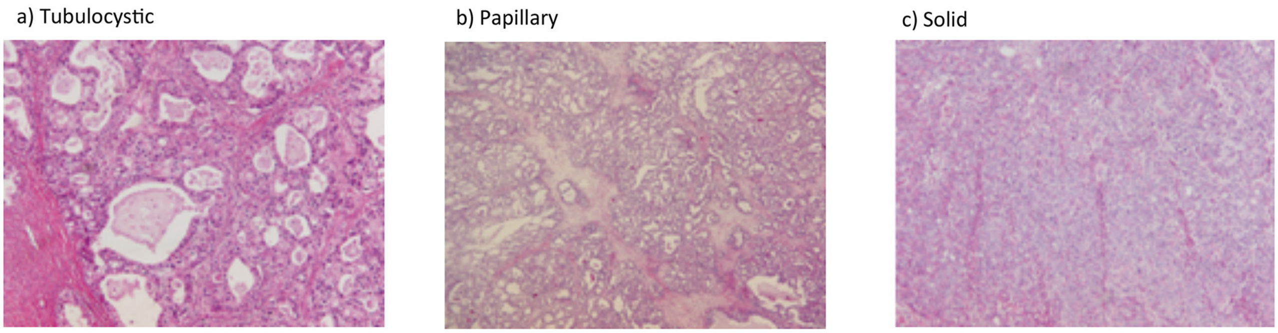

Supplementary Figure S6: Architectural patterns in ovarian clear cell carcinoma.

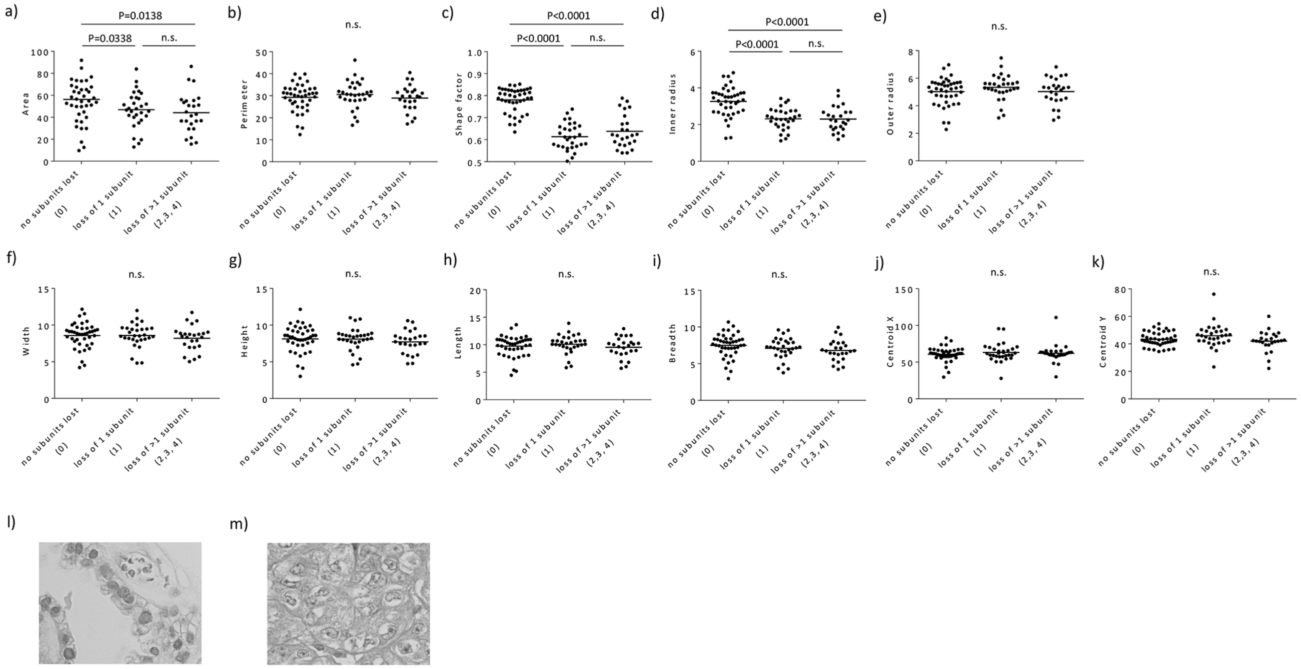

Supplementary Figure S7: Integrated morphometric analysis of nuclear shape and remaining measurements. a. Width, **b.** height, **c.** length, **d.** breadth, **e.** centroid x and **f.** centroid y. The bars represent the means for each value.

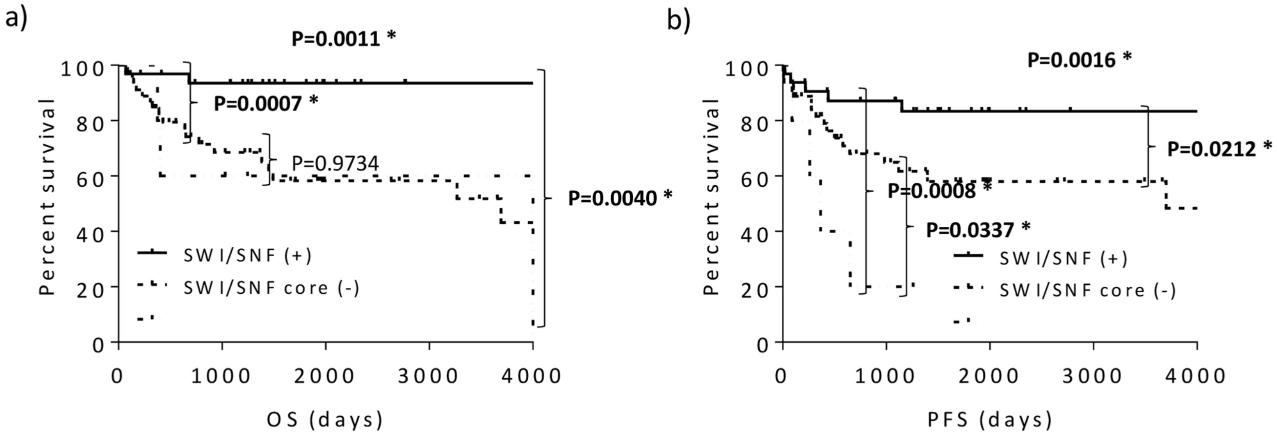

Supplementary Figure S8: Kaplan-Meier curves for overall a. and progression-free survival b. based on expression status of SWI/SNF complex core subunits (BAF250a, BAF250B, BRG1 and BRM). Cases lacking SWI/SNF non-core and core subunits showed significantly worse prognosis compared with cases expressing all SWI/SNF components.

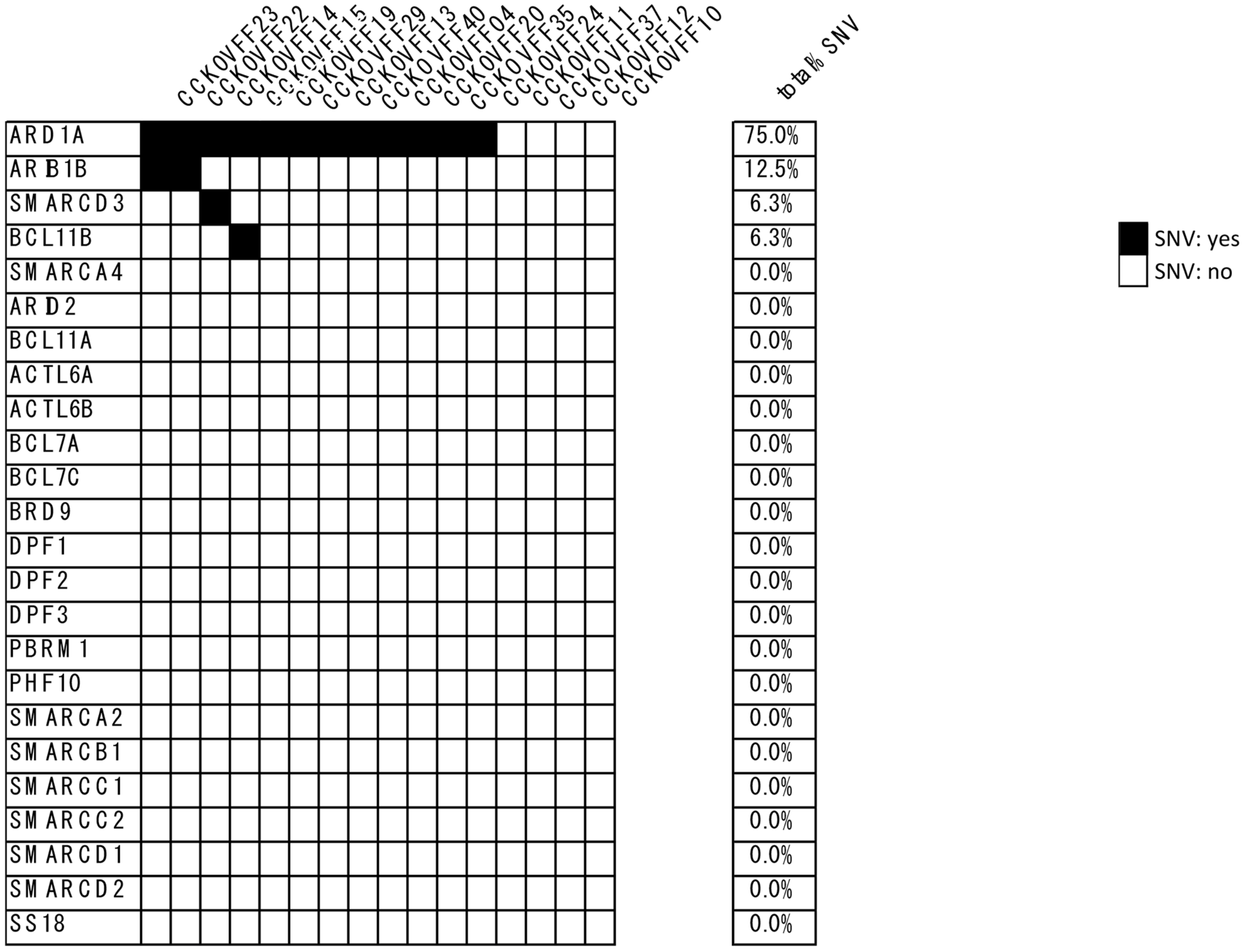

Supplementary Figure S9: Heatmap showing the main results for the mutations detected in the SWI/SNF complex. Each row represents one gene, and each column represents one sample. Black indicates a mutated sample, as determined by the comparison of single nucleotide variants between cancer samples and their normal counterparts. White indicates no mutation. The genes are ordered based on the frequency of the mutations detected.

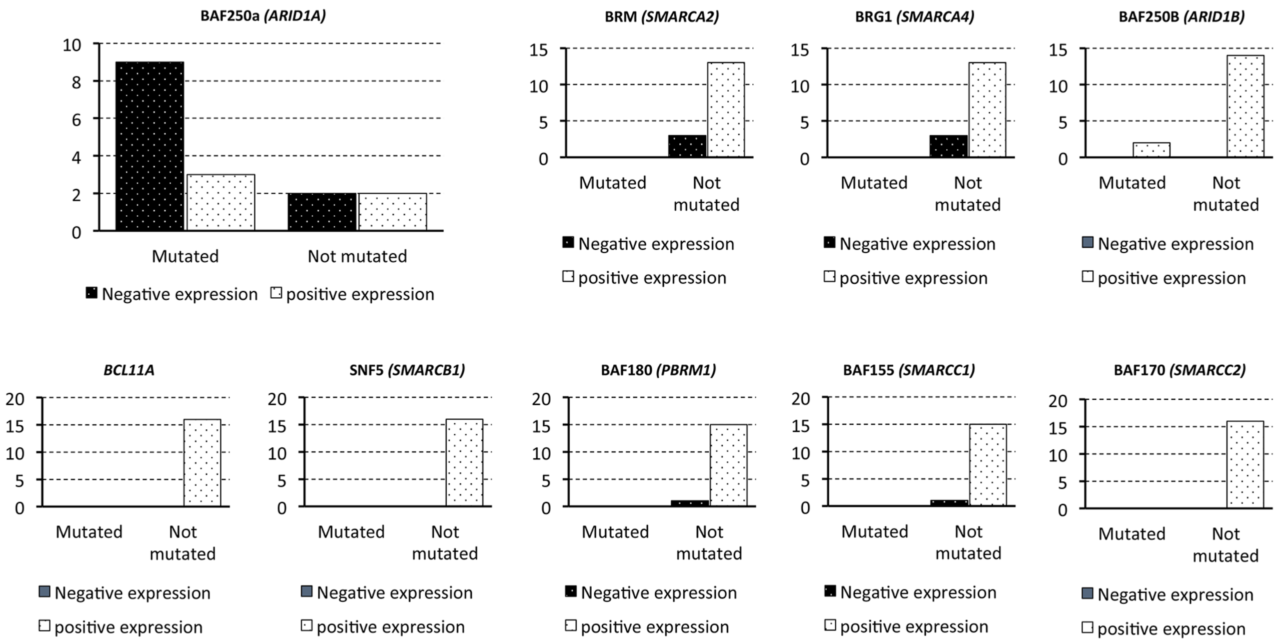

Supplementary Figure S10: Relationship between mutational and expression status.

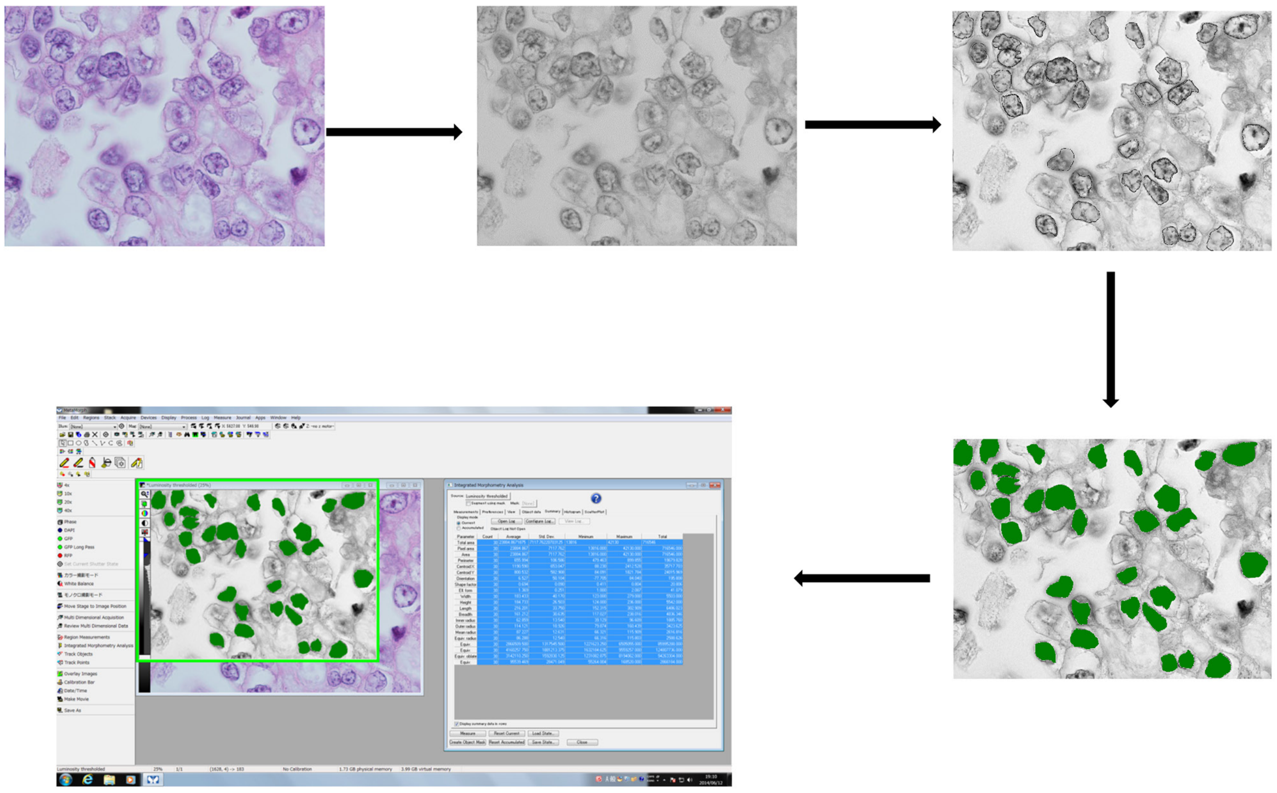

Supplementary Figure S11: Scheme for the analysis of nuclear shape using MetaMorph software.

Supplementary Table S1: Percentage of cases showing loss of SWI/SNF complex subunits in different types of ovarian cancer

Molecule	CCC (n)	CCC (%)	EAC (n)	EAC (%)	SAC (n)	SAC (%)	MAC (n)	MAC (%)	All types (n)	All types (%)
**SWI/SNF complex**	49/82	**59.76**	13/28	46.43	7/20	35	6/22	27.27	75/152	**49.34**
**BAF250a**	38/82	46.34	3/28	10.71	0/20	0.00	0/22	0.00	41/152	26.97
**BRM**	13/82	15.85	6/28	21.43	5/20	25.00	1/22	22.73	25/152	16.45
**BRG1**	9/82	10.98	0/28	0.00	3/20	15.00	2/22	13.64	14/152	9.21
**BAF170**	0/82	0.00	4/28	14.29	2/20	10.00	3/22	13.64	9/152	5.92
**BAF155**	4/82	4.88	2/28	7.14	0/20	0.00	1/22	4.55	7/152	4.61
**BAF180**	4/82	4.88	2/28	7.14	0/20	0.00	0/22	0.00	6/152	3.95
**BAF250B**	0/82	0.00	2/28	7.14	0/20	0.00	2/22	9.09	4/152	2.63
**SNF5**	2/82	2.44	2/28	7.14	0/20	0.00	0/22	0.00	4/152	2.63
**BCL11A**	2/82	2.44	0/28	0.00	0/20	0.00	0/22	0.00	2/152	1.32

Supplementary Table S2: 58 probes which overexpressed in SWI/SNF complex negative cases related to positive group.

Probe	Gene symbol	Probe	Gene symbol	Probe	Gene symbol
1569112_at	SLC44A5	218662_s_at	NCAPG	209398_at	HIST1H1C
212023_s_at	MKI67	207206_s_at	ALOX12	240253_at	NA
203127_s_at	SPTLC2	223232_s_at	CGN	1556300_s_at	SIM1
202671_s_at	PDXK	219978_s_at	NUSAP1	202503_s_at	KIAA0101
223700_at	MND1	204624_at	ATP7B	212801_at	CIT
214390_s_at	BCAT1	1568574_x_at	SPP1	207080_s_at	PYY
227952_at	NA	206794_at	ERBB4	206400_at	NA
202954_at	UBE2C	229352_at	NA	204044_at	QPRT
229080_at	EMID2	242414_at	QPRT	206172_at	IL13RA2
209035_at	MDK	220443_s_at	VAX2	209437_s_at	SPON1
212218_s_at	FASN	220324_at	LINC00472	224839_s_at	GPT2
208886_at	H1F0	212022_s_at	MKI67	235763_at	SLC44A5
35148_at	TJP3	206696_at	GPR143	219995_s_at	ZNF750
205691_at	SYNGR3	219976_at	HOOK1	241426_at	CEP44
207307_at	HTR2C	238983_at	NSUN7	209436_at	SPON1
218755_at	KIF20A	206857_s_at	FKBP1B	213994_s_at	SPON1
211682_x_at	UGT2B28	231850_x_at	CEP44	213993_at	SPON1
222586_s_at	OSBPL11	228334_x_at	CEP44	214053_at	ERBB4
213467_at	RND2	223541_at	HAS3		
223500_at	CPLX1	233498_at	ERBB4		

Supplementary Table S3: Categorical analysis of 58 probes which show overexpressing in SWI/SNF negative group related to positive group using The Database for Annotation, Visualization and Integrated Discovery (DAVID)

Category	Term	p value
SP_PIR_KEYWORDS	pyridoxal phosphate	4.67E-04
GOTERM_BP_FAT	**GO:0022403~cell cycle phase ***	5.15E-04
GOTERM_BP_FAT	**GO:0000279~M phase ***	0.001286
SP_PIR_KEYWORDS	Transferase	0.001689
GOTERM_BP_FAT	**GO:0022402~cell cycle process ***	0.00256
GOTERM_MF_FAT	GO:0016769~transferase activity, transferring nitrogenous groups	0.002607
GOTERM_BP_FAT	GO:0006323~DNA packaging	0.003103
GOTERM_MF_FAT	GO:0019842~vitamin binding	0.004298
GOTERM_BP_FAT	**GO:0008283~cell proliferation ***	0.004372
GOTERM_MF_FAT	GO:0030170~pyridoxal phosphate binding	0.008255
GOTERM_MF_FAT	GO:0070279~vitamin B6 binding	0.008255
GOTERM_BP_FAT	GO:0045785~positive regulation of cell adhesion	0.009907
SP_PIR_KEYWORDS	**mitosis ***	0.010106
GOTERM_BP_FAT	**GO:0007049~cell cycle ***	0.011838
GOTERM_BP_FAT	**GO:0000278~mitotic cell cycle ***	0.013363
SP_PIR_KEYWORDS	Nucleosome	0.016982
GOTERM_BP_FAT	**GO:0000280~nuclear division ***	0.017524
GOTERM_BP_FAT	**GO:0007067~mitosis ***	0.017524
GOTERM_BP_FAT	**GO:0000087~M phase of mitotic cell cycle ***	0.018377
PIR_SUPERFAMILY	PIRSF038219:histone H1	0.019314
GOTERM_BP_FAT	GO:0048285~organelle fission	0.019475
INTERPRO	IPR005819:Histone H5	0.021939
GOTERM_MF_FAT	GO:0048037~cofactor binding	0.024901
GOTERM_BP_FAT	GO:0007017~microtubule-based process	0.025247
SP_PIR_KEYWORDS	**cell cycle ***	0.02582
SP_PIR_KEYWORDS	**cell division ***	0.026601
SMART	SM00526:H15	0.030424
SP_PIR_KEYWORDS	rna editing	0.031313
GOTERM_BP_FAT	**GO:0007076~mitotic chromosome condensation***	0.032199
INTERPRO	IPR005818:Histone H1/H5	0.033923
UP_SEQ_FEATURE	region of interest:Globular	0.036271
GOTERM_BP_FAT	**GO:0051301~cell division***	0.037345
SP_PIR_KEYWORDS	developmental protein	0.040639
INTERPRO	IPR004839:Aminotransferase, class I and II	0.041045
GOTERM_BP_FAT	GO:0048732~gland development	0.045048
GOTERM_BP_FAT	GO:0030155~regulation of cell adhesion	0.046255
SP_PIR_KEYWORDS	acetylation	0.049426

* terms related to cell cycle and cell proliferation

Supplementary Table S4: Univariate and multivariate analyses for overall survival rate

OS			Univariate Analysis				Multivariate Analysis			
**Variable**	**subsets**	**N**	**HR**	**95% CI**		**P value**	**HR**	**95% CI**		**P value**
				**Lower**	**Upper**			**Lower**	**Upper**	
**Age**	<55y	75	1.3233	0.7066	2.4785	0.3815				
	≥55y	61								
**FIGO stage**	I & II	88	6.0998	3.0999	12.0026	******<0.0001**	3.4554	1.4791	8.0724	****0.0042**
	III & IV	48								
**Residual tumor**	Complete excision	109	4.4910	2.4173	8.3438	******<0.0001**	1.5884	0.7446	3.3883	0.2313
	Residual tumor >1cm	27								
**Tumor Size**	<10cm	42	1.7761	0.8166	3.8632	0.1474				
	≥10cm	94								
**Thromboembolism**	Absence	121	3.2576	1.5294	6.9389	****0.0022**	1.2805	0.5638	2.9080	0.5547
	Presence	15								
**SWI/SNF complex**	Number of negative subunit (0, 1)	107	5.3561	2.8333	10.1253	******<0.0001**	3.3577	1.7016	6.6254	****0.0005**
	Number of negative subunit (2, 3)	29								
**BAF250a**	Positive expression	75	1.3472	0.7233	2.5092	0.3477				
	Negative expression	61								

Univariate and Multivariate analyses were done using Cox Regression Model
* p<0.05
** p<0.005
*** p <0.0005
**** p<0.0001

Supplementary Table S5: Univariate and multivariate analyses for progression-free survival rate

PFS			Univariate Analysis				Multivariate Analysis			
Variable	subsets	N	HR	95% CI		P value	HR	95% CI		P value
				Lower	Upper			Lower	Upper	
**Age**	<55y	75	1.1550	0.6399	2.0850	0.6325				
	≥55y	61								
**FIGO stage**	I & II	88	7.4504	3.8828	14.2962	******<0.0001**	2.1692	1.4493	3.2573	******<0.0001**
	III & IV	48								
**Residual tumor**	Complete excision	109	4.9298	2.7104	8.9666	******<0.0001**	1.2034	0.8425	1.7182	0.3090
	Residual tumor >1cm	27								
**Tumor Size**	<10cm	42	1.3966	0.6913	2.8214	0.3519				
	≥10cm	94								
**Thromboembolism**	Absence	121	3.3814	1.6579	6.8963	****0.0008**	1.1161	0.7628	1.6340	0.5710
	Presence	15								
**SWI/SNF complex**	Number of negative subunit (0, 1)	107	4.3192	2.3878	7.8128	******<0.0001**	2.5707	1.3717	4.8309	****0.0030**
	Number of negative subunit (2, 3)	29								
**BAF250a**	Positive expression	75	0.9241	0.5113	1.6701	0.7938				
	Negative expression	61								

Univariate and Multivariate analyses were done using Cox Regression Model
* p<0.05
** p<0.005
*** p <0.0005
**** p<0.0001

Supplementary Table S6: Matching between immunoreactivity and mutation status in SWI/SNF complex subunits

Molecule	No mutations in positively stained samples	Mutations in positively stained samples	No mutations in negatively stained samples	Mutations in negatively stained samples	Sensitivity	Specificity	Accuracy
**BAF250a**	2	3	2	9	75.0	50.0	68.8
**BAF250B**	14	2	0	0	_	100	87.5
**BRM**	13	0	3	0	_	81.3	81.3
**BRG1**	13	0	3	0	_	81.3	81.3
**BAF155**	15	0	1	0	_	93.8	93.8
**BAF170**	16	0	0	0	_	100	100
**BAF180**	15	0	1	0	_	93.8	93.8
**SNF5**	16	0	0	0	_	100	100
**BCL11A**	16	0	0	0	_	100	100

Supplementary Table S7: Antibodies used in immunohistochemistry

	Molecule	Antibody name	Dilution rate	Company	Code	Host	Clonality
**1**	HNF1B	HNF1β (C-20)	1:100	Santa Cruz Biotechnology, Santa Cruz, CA, USA	sc-7411	Goat	Polyclonal
**2**	ERα	Mouse anti-human estrogen receptor alpha	1:100	AbD SeroTec, Oxford, UK	MCA1799T	Mouse	Monoclonal
**3**	P53	Monoclonal Mouse Anti-Human p53 Protein Clone DO-7	Ready to use	Dako, Denmark	M 7001	Mouse	Monoclonal
**4**	pMAPK	Phospho-p44/42 MAPK (Erk1/2) (Thr202/Tyr204) (D13.14.4E) XP® Rabbit mAb	1:400	Cell Signaling, Danvers, MA, USA	4370	Rabbit	Monoclonal
**5**	pAKT	Phospho-Akt (Ser473) (D9E) XP® Rabbit mAb	1;50	Cell Signaling, Danvers, MA, USA	4060	Rabbit	Monoclonal
**6**	Ki67	Monoclonal Mouse Anti-Human Ki-67 Antigen clone MIB-1	1:200	Dako, Denmark	M 7240	Mouse	Monoclonal
**7**	BAF250a (ARID1A)	Anti-ARID1A antibody produced in rabbit	1:300	Sigma Aldrich, St Louis, MO, USA	HPA005456	Rabbit	Polyclonal

(*Continued*)

	Molecule	Antibody name	Dilution rate	Company	Code	Host	Clonality
**8**	BRG1	BRG1 antibody (G-7)	1:100	Santa Cruz Biotechnology, Santa Cruz, CA, USA	sc-17796	Mouse	Monoclonal
**9**	BRM	Anti-SMARCA2 antibody produced in rabbit	1:200	Sigma Aldrich, St Louis, MO, USA	HPA029981	Rabbit	Polyclonal
**10**	BAF180 (PBRM1)	Anti-PBRM1 antibody produced in rabbit	1:200	Sigma Aldrich, St Louis, MO, USA	HPA015629	Rabbit	Polyclonal
**11**	BAF250B (ARID1B)	Anti-ARID1B antibody	1:100	Abcam, Cambridge, MA, USA	Ab57461	Mouse	Monoclonal
**12**	BAF155	BAF155 (DXD7)	1:100	Santa Cruz Biotechnology, Santa Cruz, CA, USA	sc-32763	Mouse	Monoclonal
**13**	BAF170	BAF170 (E-6)	1:200	Santa Cruz Biotechnology, Santa Cruz, CA, USA	sc-17838	Mouse	Monoclonal
**14**	SNF5	Purified Mouse Anti-BAF47	1:200	BD biosciences, San Jose, CA, USA	612110	Mouse	Monoclonal
**15**	BCL11A	BCL11A monoclonal antibody (M03), clone 3D9	1:100	Abnova, Taipei, Taiwan	H00053335-M03	Mouse	Monoclonal
